# Whole-Genome Transformation Promotes tRNA Anticodon Suppressor Mutations under Stress

**DOI:** 10.1128/mBio.03649-20

**Published:** 2021-03-23

**Authors:** Quinten Deparis, Jorge Duitama, Maria R. Foulquié-Moreno, Johan M. Thevelein

**Affiliations:** a Laboratory of Molecular Cell Biology, Institute of Botany and Microbiology, KU Leuven, Belgium; b Center for Microbiology, VIB, Leuven-Heverlee, Flanders, Belgium; c Systems and Computing Engineering Department, Universidad de los Andes, Bogotá, Colombia; d NovelYeast bv, Open Bio-Incubator, Erasmus High School, Brussels (Jette), Belgium; Wake Forest; Yonsei University

**Keywords:** anticodon, thermotolerance, tRNA, whole-genome transformation, yeast

## Abstract

tRNAs are encoded by a large gene family, usually with several isogenic tRNAs interacting with the same codon. Mutations in the anticodon region of other tRNAs can overcome specific tRNA deficiencies. Phylogenetic analysis suggests that such mutations have occurred in evolution, but the driving force is unclear. We show that in yeast suppressor mutations in other tRNAs are able to overcome deficiency of the essential *TRT2*-encoded tRNA^Thr^_CGU_ at high temperature (40°C). Surprisingly, these tRNA suppressor mutations were obtained after whole-genome transformation with DNA from thermotolerant Kluyveromyces marxianus or Ogataea polymorpha strains but from which the mutations did apparently not originate. We suggest that transient presence of donor DNA in the host facilitates proliferation at high temperature and thus increases the chances for occurrence of spontaneous mutations suppressing defective growth at high temperature. Whole-genome sequence analysis of three transformants revealed only four to five nonsynonymous mutations of which one causing *TRT2* anticodon stem stabilization and two anticodon mutations in non-threonyl-tRNAs, tRNA^Lys^_CUU_ and tRNA^eMet^_CAU_, were causative. Both anticodon mutations suppressed lethality of *TRT2* deletion and apparently caused the respective tRNAs to become novel substrates for threonyl-tRNA synthetase. Liquid chromatography-tandem mass spectrometry (LC-MS/MS) data could not detect any significant mistranslation, and reverse transcription-quantitative PCR results contradicted induction of the unfolded protein response. We suggest that stress conditions have been a driving force in evolution for the selection of anticodon-switching mutations in tRNAs as revealed by phylogenetic analysis.

## Abstract (precis)

In this work, we have identified for the first time the causative elements in a eukaryotic organism introduced by applying whole-genome transformation and responsible for the selectable trait of interest, i.e., high temperature tolerance. Surprisingly, the whole-genome transformants contained just a few single nucleotide polymorphisms (SNPs), which were unrelated to the sequence of the donor DNA.

## INTRODUCTION

tRNAs and aminoacyl-tRNA synthetases (aaRSs) have recently become of increasing interest due to the discovery of novel regulatory mechanisms and roles for these molecules (1). Saccharomyces cerevisiae uses 61 different codons, which are recognized by 42 tRNAs encoded by 275 tRNA genes in the yeast genome. For some anticodons there are between 2 and 16 redundant tRNA genes encoding isoacceptor-tRNAs, while for some of the 61 potential anticodons there is only one or even no dedicated tRNA gene encoded in the genome, requiring alternative mechanisms for their efficient translation (2). Also, not all codons are used equally in orfeomes, which is referred to as “codon bias.” The use of preferred codons affects translation rates (3) and (cotranslational) folding of proteins (4, 5), as well as translational accuracy (6). Species-specific genes often show aberrant codon usage compared to evolutionarily conserved genes, which can have a profound impact on expression of the gene product (7).

Isoacceptor tRNAs are differentially expressed depending on environmental conditions and might play different roles under the different conditions as suggested by results obtained with a complete yeast tRNA deletion library (8). Deletion of specific members of an isoacceptor tRNA gene family results in different and sometimes even opposing resistance phenotypes under stress conditions. More frequently used codons tend to have a higher number of isoacceptor tRNA genes (9). On the other hand, for some codons there is no gene encoding a tRNA with the complementary anticodon (8). This deficiency can be solved in several ways: use of a tRNA with a similar anticodon through wobble interactions, noncanonical base pairing, a mutation in the anticodon sequence of the gene encoding another tRNA (usually a multicopy isoacceptor tRNA), or posttranscriptional modification of a tRNA.

Whereas the first and second nucleotide of a codon normally require precise Watson-Crick base pairing for efficient translation, the interaction between the third base of the codon and the first base of the anticodon is less strict (10, 11). Nonetheless, these interactions also follow a defined set of rules, limiting the number of possible codon-anticodon interactions (12). Wobble interactions involve non-Watson-Crick base pairing at the third nucleotide of the codon and are achieved by tRNA modifications at position 34 or even at more distal sites in the tRNA (13, 14). Adenosines at position 34 are especially prone to modification. Deamination of adenosine into inosine by adenosine deaminase or spontaneous conversion allows for wobble interactions with adenine, cytosine and uracil (15). In addition to I⋅A, I⋅C, and I⋅U base pairing, noncanonical G⋅U base pairing is the most frequently observed mismatch at position 34 in tRNAs (12, 16). Since some codons do not have a gene present in the genome encoding a tRNA with their cognate anticodon, base pairing with inosine and G⋅U base pairing are essential for proper translation (17, 18). tRNA modifications and wobble base pairing are especially important in mitochondria and chloroplasts since they generally do not contain multiple isoacceptor tRNAs for a single anticodon and also lack a dedicated tRNA for many more codons compared to the nucleus (19–21). Wobble base pairing with other tRNAs has been observed to modulate expression levels of gene products even for codons which have a dedicated tRNA gene (22–24). Since tRNAs do not only play a role in supplying building blocks for protein synthesis, but also have regulatory roles, their cellular levels are tightly regulated (1, 25). Hypomodified or unstable tRNAs are therefore quickly degraded by the rapid tRNA degradation (RTD) pathway and the *MET22*-dependent decay pathway (26–28).

Yeast can also suppress tRNA deficiencies by generating mutations in the anticodon sequence of other tRNA genes creating the same anticodon as that of the defective tRNA. Such mutations usually occur in near-cognate anticodons. Deletion of the unique tRNA gene *HSX1*, encoding tRNA^Arg^_CCU_, caused a strong growth defect and upregulation of the unfolded protein response (UPR) and enhanced expression of several chaperones indicated that the *hsxΔ* strain suffered from proteotoxic stress. After adaptive evolution, spontaneously generated mutants were obtained that displayed faster growth. Whole-genome sequencing of several of these evolved strains revealed mutations at position 34 in the anticodon in a copy of one of the 11 tRNA^Arg^_UCU_ isoacceptor genes (29), which alleviated the accumulation of mistranslated proteins in the cytosol. tRNA genes are known to mutate at rates 7 to 10 times higher than protein coding regions by transcription-associated mutagenesis (30). In this case, the strong growth defect caused by *hsx1Δ* apparently served as the driving force for generation and selection of the tRNA suppressor mutations. Comparative phylogenomic analysis suggested the existence of such anticodon switching events throughout evolution (29, 31). However, the driving force for generation and selection of such mutations in nature has remained unclear.

Whole-genome transformation (WGT) has been used in prokaryotes and eukaryotes to transfer phenotypic traits displayed by the genomic DNA (gDNA) donor strain to the recipient host strain. Donor and host strain can belong to the same or to different species. In bacteria, WGT has been used frequently to identify antibiotic resistance mutations by transformation of the gDNA from the donor antibiotic-resistant strain into an antibiotic-sensitive strain from the same species, followed by comparative whole-genome sequence analysis of the gDNA from donor and transformant strain. This has been performed with clinical isolates of Streptococcus pneumoniae, which resulted in identification of key variants in *Pbp2x* responsible for their penicillin resistance (32). Similarly, other bacterial species have been transformed by WGT (33–35). In these cases, the close sequence relatedness between the strains resulted in transfer of only single nucleotide polymorphisms (SNPs), including causative SNPs, by homologous recombination (36). An S. cerevisiae strain was transformed with the DNA of the xylose-assimilating yeast Pichia stipitis, enabling the transformant to metabolize xylose (37). Since intact enzyme activities are required for xylose utilization capacity, larger fragments of donor gDNA must have been transferred and stably inserted into the S. cerevisiae host genome. In addition, RAPD [random(ly) amplified polymorphic DNA] analysis confirmed the presence of large *P. stipitis* genomic DNA fragments. Similarly, transfer of wild rice cultivar traits by WGT into a cultivar of domesticated Oryza sativa was accompanied by integration and rearrangements of large genomic fragments (38). In both cases, the precise causative genetic modifications have remained unclear. These observations appear to have led to the general assumption that in eukaryotes WGT with gDNA from different species leads to insertion and rearrangements of large heterologous genomic DNA fragments for which identification of the precise causative element(s) is highly challenging.

Microbial thermotolerance is an important trait in many natural environments, and it is also important in many industrial biotechnology applications with yeast. Yeast fermentation in commercial-scale facilities generates large amounts of heat, which requires constant cooling of the fermenters. Inadequate cooling and/or mixing of the broth can lead to temperature gradients, especially at high environmental temperatures, which can compromise yeast fermentation performance (39). Many factors have been identified that are required for yeast thermotolerance (40), including (t)RNA processing (41, 42). Genetic modification of such factors generally leads to a drop in thermotolerance. On the other hand, much fewer genetic modifications have been identified that result in improved thermotolerance (43). Generally, evaluation of thermotolerance has been carried out with growth assays, either on solid nutrient plates or in liquid cultures. Much less attention has been devoted to thermotolerance of yeast performance under conditions mimicking industrial fermentations.

Here, we have used WGT for improvement of fermentation performance at high temperature of a haploid segregant of an industrial bioethanol production S. cerevisiae strain. gDNA from the thermotolerant nonconventional yeast species Kluyveromyces marxianus and *Ogataea* (*Hansenula*) *polymorpha* has been used as donor DNA. After selection of thermotolerant transformants, whole-genome sequence analysis of three independent strains surprisingly revealed a very low number of nonsynonymous SNPs, of which in each case a mutation in a tRNA gene that stabilized or created a Thr^CGU^ anticodon containing tRNA, was causative. Liquid chromatography-tandem mass spectrometry (LC-MS/MS) data indicate absence of any significant mistranslation in the anticodon mutated strains. This suggests that the mutated tRNAs apparently function *in vivo* as the substrates for threonyl-tRNA synthetase (ThrRS). Our results suggest that stress conditions can act as driving force for compensating tRNA mutations, explaining the plasticity of the genomic tRNA landscape as suggested by phylogenomic analysis.

## RESULTS

### Isolation of thermotolerant transformants of ER18A HPH by WGT.

**FIG 1:**
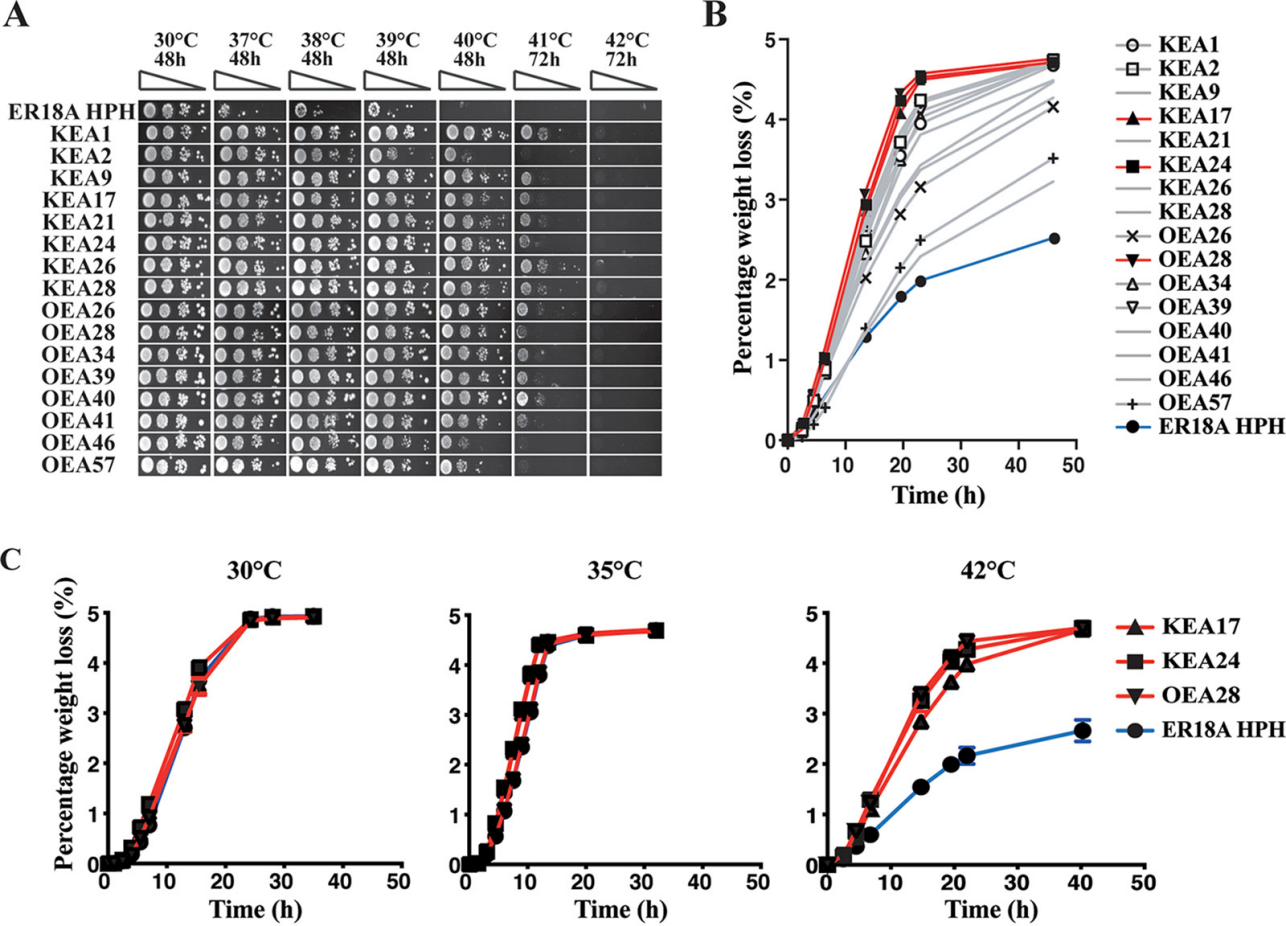
Growth on solid nutrient medium and fermentation under semianaerobic conditions of whole-genome transformants at different temperatures. (A) Thermotolerance of growth on solid YPD medium. ER18A HPH, host strain; KEA strains, transformants with K. marxianus genomic DNA; OEA strains, transformants with *O. polymorpha* genomic DNA. Tenfold dilutions of stationary-phase cultures (OD 0.5) were spotted and allowed to grow at different temperatures for 2 or 3 days as indicated. (B) Thermotolerance of fermentations (60 ml of YPD with 10% glucose) under semianaerobic conditions at 42°C. Blue, host strain ER18A HPH; red, three selected transformants with the highest thermotolerance submitted for whole-genome sequencing. Symbols: solid triangles, KEA17; solid boxes, KEA24; solid inverted triangles, OEA28. Gray with symbols: same transformants as in panel A, which were also submitted for whole-genome sequencing. Open circles, KEA1; open boxes, KEA2; open triangles, KEA28, ×, OEA26; open inverted triangles, OEA34; open diamonds, OEA39; +, OEA57. Gray, no symbol: remaining transformants as in panel A. Error bars represent the standard deviations (SD) of three biological repeats. (C) Fermentation of the three selected transformants at 30, 35, and 42°C. (Other conditions are as described in panel B.) All fermentations were performed with three biological repeats.

ER18A, a haploid derivative of the first-generation bioethanol strain ER (Fermentis, a division of Lesaffre) has a relatively low maximal growth temperature on solid nutrient plates (39°C; Fig. 1A) and an incomplete fermentation at 42°C (Fig. 1B). The hygromycin B resistance gene *HPH* was inserted in the genome at the position 867,910 in chromosome IV to easily distinguish the strain from possible thermotolerant contaminants. WGT with gDNA from different sources was used to isolate independent transformants displaying superior growth at 40°C on solid nutrient plates. Transformations were performed with only water (blank control), ER18A gDNA, gDNAs of other randomly selected S. cerevisiae strains, gDNA of two nonconventional, nonthermotolerant yeast species (Zygosaccharomyces rouxii and Z. bailii), and gDNAs of two nonconventional, thermotolerant yeast species (K. marxianus and *O. polymorpha*). Only WGT with gDNA of the two thermotolerant yeast species resulted in transformant colonies visible after 3 to 5 days at 40°C, that remained stably thermotolerant after seven consecutive rounds of incubation under nonselective conditions (30°C). Selection at higher temperatures did not result in any transformants. Occasionally, WGT with gDNA of other strains resulted in very few colonies, but these strains never retained the thermotolerant phenotype after subculturing under nonselective conditions. Such unstable thermotolerant strains were also obtained occasionally with gDNA from K. marxianus and *O. polymorpha*. The stable WG transformants obtained with gDNA of K. marxianus were named KEA (*Kluyveromyces* ER18A) and with gDNA from *O. polymorpha* OEA (*Ogataea* ER18A). About 100 of these transformants together with the parent strain ER18A HPH were evaluated for thermotolerance using growth on solid nutrient medium with yeast extract peptone (YP) and 2% glucose incubated at different temperatures, ranging from 30 to 42°C, and also in small-scale fermentations with YP and 10% glucose at 42°C. The results obtained for a representative sample are shown in Fig. 1A and B. All stable WG transformants showed various degrees of higher thermotolerance compared to the parent strain ER18A HPH. From the 100 strains evaluated, the three most-thermotolerant strains in the small-scale fermentation assay, KEA17, KEA24, and OEA28, as well as the parent strain ER18A HPH, were again evaluated in small-scale fermentations with YP and 10% glucose incubated at 30, 35, and 42°C (Fig. 1C). All strains showed the same fermentation performance at 30 and 35°C, excluding a general growth and/or fermentation advantage of the WG transformants compared to the parent strain. However, the three WG transformants showed much higher fermentation performance at 42°C compared to the parent strain ER18A HPH. On the other hand, none of the WG transformants showed any growth on solid nutrient medium at 42°C. Moreover, the thermotolerance for growth on solid nutrient medium was poorly correlated with thermotolerance of fermentation in liquid medium, except that very poor growth on plates was always correlated with very poor fermentation performance in liquid medium. The correct identity of the three superior transformants was confirmed by presence of the HPH marker, verification of the mating type and the ploidy, and by scoring a few SNPs specific for the ER18A genetic background.

### Identification of SNPs in the three most-thermotolerant transformants.

The three most-thermotolerant WG transformants (KEA17, KEA24, and OEA28), as well as the parent strain ER18A HPH, were submitted to WG sequence analysis (BGI, Hong Kong). This revealed the absence of any detectable heterologous DNA fragments or even SNPs from either K. marxianus or *O. polymorpha*. Instead, a very small number of new SNPs, including just five, four and five nonsynonymous, promoter or tRNA SNPs, was found in the transformants KEA17, KEA24, and OEA28, respectively (Table 1). Comparative sequence analysis of these DNA regions confirmed that none of these SNPs were present in the genome of K. marxianus or O. polymorpha. Furthermore, a duplication of chromosome III had occurred in both K. marxianus transformants but not in strain OEA28. Remarkably, in each of the three WG transformants one of the mutations was present in a tRNA encoding gene, which was different in the three strains (Table 1).

**TABLE 1:** Polymorphisms and chromosome duplications identified in the whole-genome transformants KEA24, KEA17, and OEA28 after whole-genome sequence analysis^*a*^

Modification	Chr.	Gene	Feature type	Base change	Amino acid change
KEA17					
CD	III	NA	NA	NA	NA
SNP	IV	*EAF1*	ORF	c.924 G>T	K308N
SNP	XI	*tK(CUU)K*	tRNA^Lys^_CUU_	c.35 T>G	NA
SNP	XII	*NHA1*	Promoter	−107 C>A	NA
SNP	XIV	*BSC5*	Promoter	−728 C>A	NA
					
KEA24					
CD	III	NA	NA	NA	NA
SNP	VII	*YHK8*	ORF	c.121 C>T	R41*
SNP	XII	*HMX1*	Promoter	−36 C>T	NA
Indel	XV	*RTT10*	Promoter	−124 AAT>AT	NA
Indel	XVI	*RPL36B*	Promoter	−329 TG>TTG	NA
SNP	XVI	*EMT2*	tRNA^Met^_CAU_	c.35 A>G	NA
					
OEA28					
SNP	IX	*MPH1*	ORF	c.2636 A>G	D879G
SNP	XI	*TRT2*	tRNA^Thr^_CGU_	c.40 C>A	NA
SNP	XIII	*ILV2*	ORF	c.138 C>T	A461V
Indel	XVI	*CET1*	Promoter	−664 CTT>CT	NA
SNP	XVI	*PIS1*	ORF	c.280 G>A	V93M

a Chr., chromosome; CD, chromosomal duplication; SNP, single-nucleotide polymorphism; ORF, open reading frame; NA, not applicable; *, nonsense mutation.

### Identification of causative SNPs in KEA17, KEA24, and OEA28.

**FIG 2:**
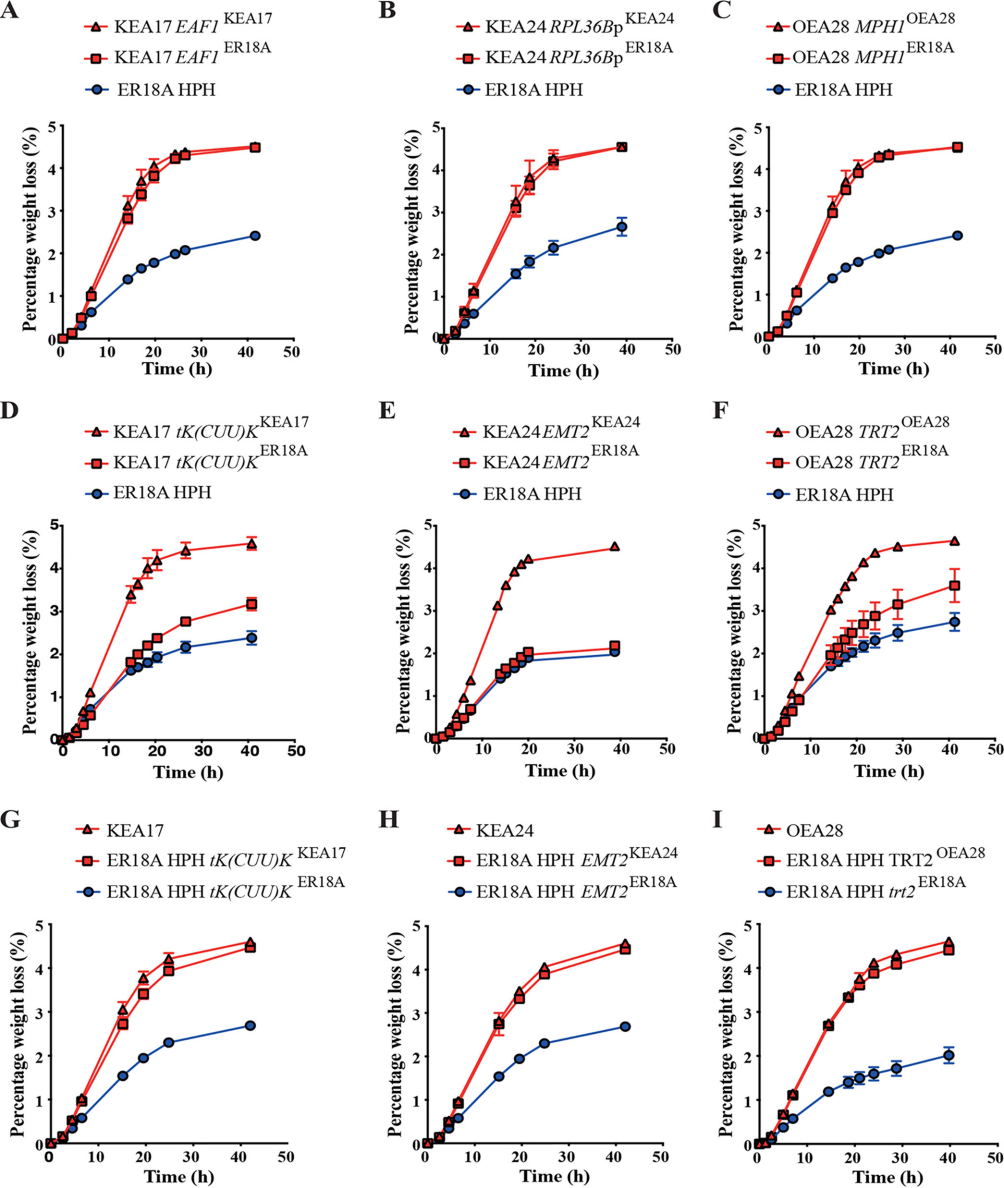
Fermentations at 42°C of the host strain ER18A HPH, the whole-genome transformants KEA17, KEA24, and OEA28, and the ER18A HPH, KEA17, KEA24, and OEA28 strains engineered for a putative causative SNP to downgrade/upgrade thermotolerance. For ER18A HPH, examples of noncausative SNPs (A to C), causative SNPs (D to F), and upgrades (G to I) are shown. Error bars represent the SD of three biological repeats. (A) KEA17 and KEA17 with *EAF1*^ER18A^. (B) KEA24 and KEA24 with *RPL36B*p^ER18A^. (C) OEA28 and OEA28 with *MPH1*^ER18A^. (D) KEA17 and KEA17 with *tK(CUU)K*^ER18A^. (E) KEA24 and KEA24 with *EMT2*^ER18A^. (F) OEA28 and OEA28 with *trt2*^ER18A^. (G) ER18A HPH with *tK(CUU)K*^KEA17^. (H) ER18A HPH with *EMT2*^KEA24^. (I) ER18A HPH with *TRT2*^OEA28^.

To identify which one of the nonsynonymous SNPs was causative for the increased thermotolerance, we reengineered for each SNP the original ER18A nucleotide in the respective transformant using CRISPR/Cas9 technology. The resulting strains were evaluated for thermotolerance in small-scale fermentations with YP and 10% glucose. The results showed that none of the reengineered strains lost their increased thermotolerance (Fig. 2A, B, and C; see also Fig. S1 in the supplemental material), except for the strains reengineered with the original nucleotide of the tRNA gene (Fig. 2D to F). Hence, the three mutated tRNA genes [*tK(CUU)K*, *EMT2*, and *TRT2*] were identified as the causative genetic element in the three most-thermotolerant WG transformants, KEA17, KEA24, and OEA28, respectively (Fig. 2D to F). This was confirmed by the reverse modification in which we engineered the three tRNA mutations in the corresponding tRNA genes of the original ER18A HPH host strain. In each case, this conferred a similar level of increased thermotolerance to the host strain (Fig. 2G to I).

10.1128/mBio.03649-20.1 FIG S1 Fermentations at 42°C of the host strain ER18A HPH, the whole-genome transformants KEA17, KEA24, and OEA28, and the KEA24, KEA17, and OEA28 strains engineered for a noncausative SNP to downgrade thermotolerance. (A) KEA17 and KEA17 with *BSC5*p^ER18A^. (B) KEA17 and KEA17 with *NHA1*p^ER18A^. (C) KEA24 and KEA24 with *HMX1*p^ER18^. (D) KEA24 and KEA24 with *RTT10*p^ER18A^. (E) KEA24 and KEA24 with *YHK8*^ER18A^. (F) OEA28 and OEA28 with *PIS1*^ER18A^. (G) OEA28 and OEA28 with *ILV2*^ER18A^. (H) OEA28 and OEA28 with *CET1*p^ER18A^. Download FIG S1, TIF file, 2.2 MB. Copyright © 2021 Deparis et al. 2021 Deparis et al. https://creativecommons.org/licenses/by/4.0/ This content is distributed under the terms of the Creative Commons Attribution 4.0 International license.

### tRNA^Thr^_CGU_ is a critical determinant of heat tolerance.

**FIG 3:**
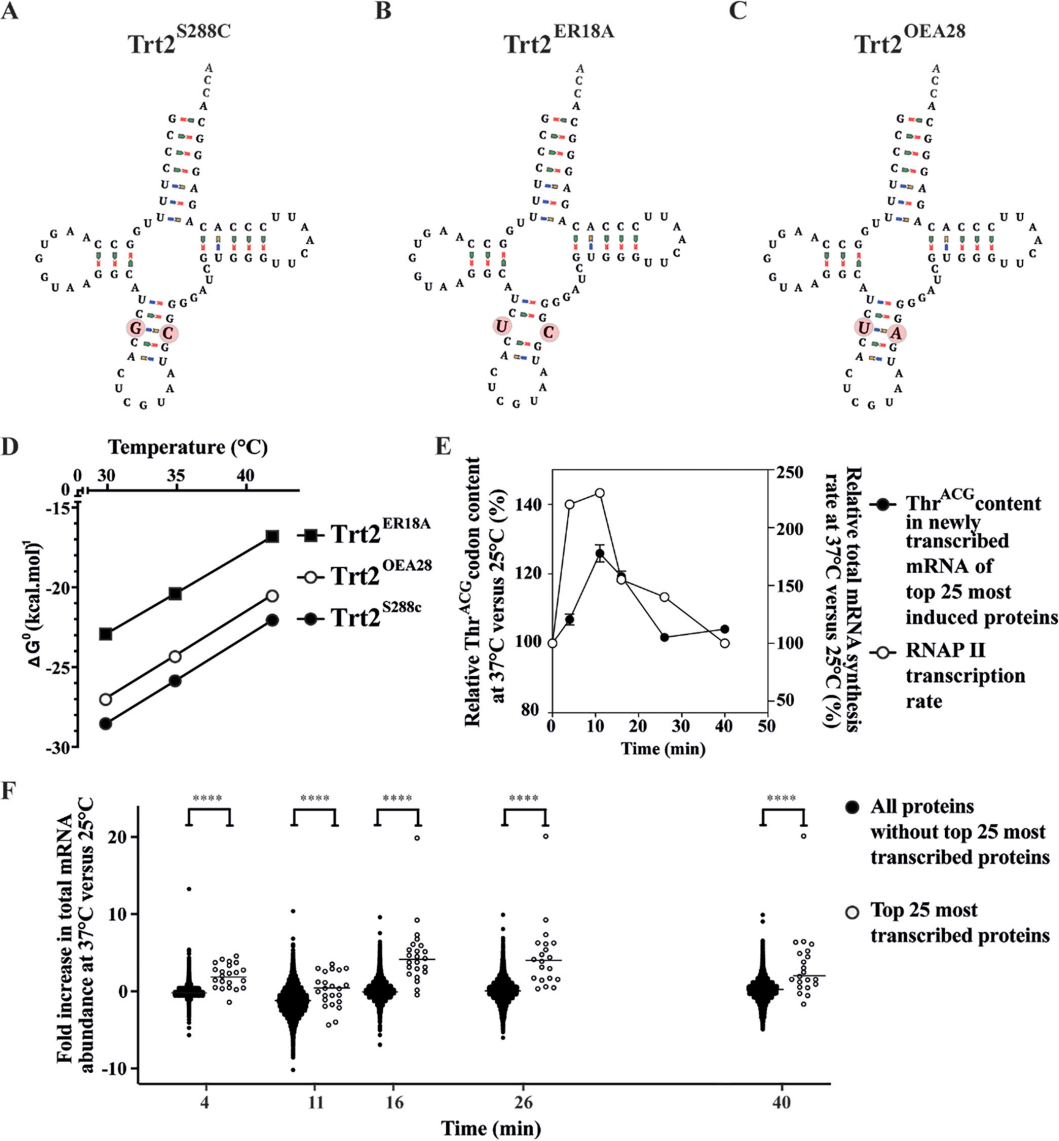
Structure of three Trt2 isoforms and their calculated stabilities at various temperatures and the possible importance of *TRT2*-encoded tRNA^Thr^_CGU_ under heat stress. Differences between the S288C, ER18A HPH, and OEA28 variants are indicated by a red circle in panels A, B, and C. (A) Trt2^S288C^. (B) Trt2^ER18A^. (C) Trt2^OEA28^. (D) Calculated Gibbs energy of folding Δ*G*^0^ (kcal/mol) of *TRT2*-encoded tRNA^Thr^_CGU_ as a function of temperature. The Gibbs energy of folding of tRNA^Thr^_CGU_ encoded by *trt2*^ER18A^ (solid boxes) is much higher than that of tRNA^Thr^_CGU_ encoded by *TRT2*^S288C^ (solid circles), whereas the tRNA^Thr^_CGU_ encoded by *TRT2*^OEA28^ (open circles) has much lower folding energy. (E) Relative Thr^ACG^ codon content in mRNA transcripts and mRNA synthesis rate upon heat treatment. Error bars represent the SD of three repeats. Solid circles, relative Thr^ACG^ codon content in newly synthesized mRNA of top 25 upregulated genes at 37°C versus 25°C; open circles, relative RNA polymerase II (RNAP) synthesis rate at 37°C versus 25°C, taken from Castells-Roca et al. (46). (F) Fold increase in total mRNA abundance upon heat treatment. Open circles, mRNA abundance levels of the top 25 most induced genes at 37°C versus 25°C; solid circles, mRNA abundance levels of all remaining proteins at 37°C versus 25°C. Statistical analysis was performed using the Kruskal-Wallis test (ns, *P* > 0.05; *, P < 0.05; **, *P* < 0.01; ***, *P* < 0.001; ****, *P* < 0.0001).

Remarkably, the outcome of the three tRNA mutations all pointed to the unique *tRNA^Thr^_CGU_ TRT2* gene as a critical determinant of thermotolerance in the ER18A HPH host strain. In S. cerevisiae, *TRT2* only comprises 72 bp in contrast to many other tRNAs that are composed of 73 bp or more. Since *TRT2* encodes a singleton tRNA, proper functioning is crucial for the cell’s survival, even under non-stressed conditions (44). The tRNA^Thr^ *TRT2* gene in strain ER18A contains a mutation, 28 G>U, compared to the standard S. cerevisiae genome, represented by the laboratory model strain S288C, which results in loss of base pairing, as opposed to the regular GC base pair in the anticodon stem (Fig. 3A and B). The loss of base pairing presumably lowers the structural stability of the tRNA at the higher temperature of 42°C, but is tolerable at 30 and 35°C, and since the tRNA^Thr^ *TRT2* gene is only present in a single copy in the genome it likely limits thermotolerance of the strain. This is supported by the nature of the causative tRNA mutation in WG transformant OEA28, in which the C40 residue that could not base pair with U28, is mutated to adenine, resulting in (noncognate, but genuine) UA base pairing (Fig. 3C).

In order to evaluate the effect of the mutation in the anticodon stem of the *tRNA^Thr^_CGU_*, we calculated the Gibbs energy of folding for the three Trt2 isoforms (Fig. 3D). These thermal stability calculations suggest that Trt2^ER18A^ had a higher Δ*G*^0^ than did Trt2^S288C^, a finding indicative of reduced stability of this variant. Trt2^OEA28^ with a suppressor mutation in the anticodon stem that restored base pairing, showed a lower Δ*G*^0^ compared to the ER18A variant, close to that of the wild-type Trt2 in the S288C reference laboratory strain, indicating higher thermal stability. It has previously been shown that mutations in tRNAs that result in an increase of Δ*G*^0^ larger than 2.65 kcal/mol usually result in defective tRNAs that are readily degraded by the RTD pathway (45).

We have also studied the requirement of the unique Thr^ACG^ codon at high temperature in more detail. It has been reported that upon a temperature shift from 25 to 37°C, RNA polymerase II expression transiently increases (46). The 25 genes with the highest transient increase in expression during the same temperature shift contained a relatively higher proportion of the unique Thr^ACG^ codon, indicating a higher requirement for the critical tRNA^Thr^_CGU_ in the mRNAs expressed at a higher temperature (Fig. 3E). In addition, the average mRNA abundance level of these 25 genes increased significantly more than the average mRNA abundance level of all proteins upon the same temperature shift, again consistent with a higher demand for the tRNA^Thr^_CGU_ at high temperature (Fig. 3F).

### Anticodon mutations in *tK(CUU)K* and *EMT2* rescue lethality of the *TRT2* deletion mutant.

**FIG 4:**
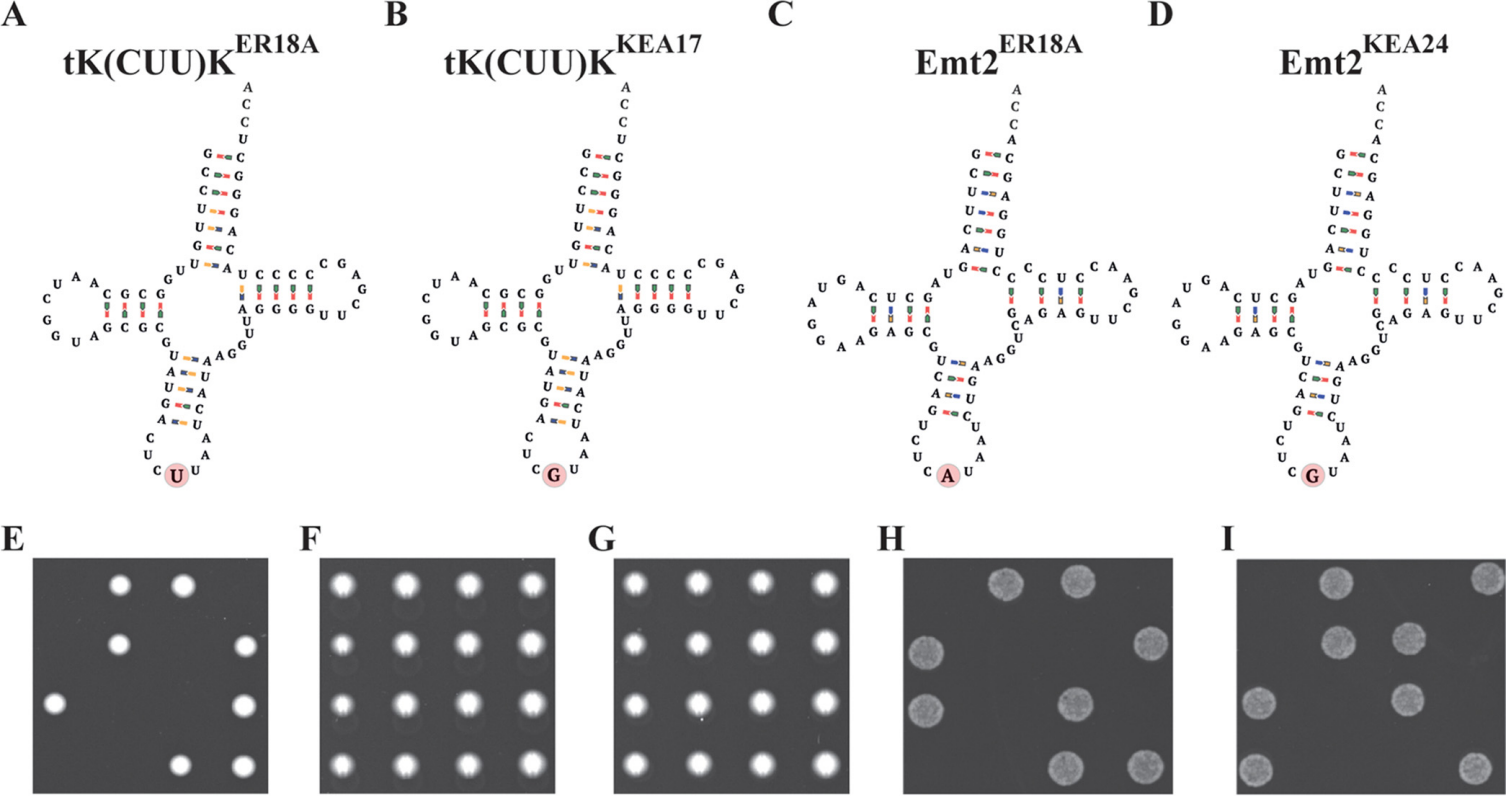
Structure of the ER18A HPH, KEA17 and KEA24 tRNA isoforms and rescue of lethality caused by deletion of the essential *TRT2* gene by expression of the mutant *tK(CUU)K*^KEA17^ allele or the mutant allele *EMT2*^KEA24^, as shown by tetrad analysis of the *TRT2/trt2*::*NatMX* diploid strain QD48. Differences between the ER18A HPH, KEA17, and KEA24 variants are indicated with a red circle in panels A, B, C, and D. (A) *tK(CUU)K*^S288C^ = *tK(CUU)K*^ER18A^. (B) *tK(CUU)K*^KEA17^. (C) *EMT2*^S288C^ = *EMT2*^ER18A^. (D) *EMT2*^KEA24^. (E to I) Tetrad analysis was performed for the indicated following strains. Rows represent complete tetrads. (E) QD48, revealing two-to-two segregation for viability/lethality. (F) QD48 *tK(CUU)K*^KEA17^/*tK(CUU)K*^KEA17^ showing suppression of *trt2Δ* lethality. (G) QD48 *EMT2*^KEA24^/*EMT2*^KEA24^ showing suppression of *trt2Δ* lethality. (H) QD48 *tK(CUU)K*^KEA17^/*tK(CUU)K*^KEA17^ in the presence of nourseothricin. (I) QD48 *EMT2*^KEA24^/*EMT2*^KEA24^ in the presence of nourseothricin. *NatMX*, encoding nourseothricin acetyltransferase, is a nourseothricin resistance marker. In the presence of nourseothricin, only *trt2*::*NatMX* segregants can grow.

In WG transformant KEA17, the anticodon of the tRNA^Lys^_CUU_ [*tK(CUU)K*] gene is mutated into the CGU anticodon of Trt2 (Fig. 4A and B). Since there are 14 copies of the tRNA^Lys^_CUU_ [*tK(CUU)K*] gene in the genome, the loss of a single copy is unlikely to create a limitation in the provision of tRNA^Lys^_CUU_. In WG transformant KEA24 a similar event happened. The anticodon of the elongator tRNA^Met^_CAU_ (*EMT2*) gene is also mutated to the CGU anticodon of Trt2 (Fig. 4C and D). Since there are five tRNA^eMet^_CAU_ gene copies in the genome, loss of a single copy does presumably also not create a limitation in the provision of tRNA^eMet^_CAU_. Consistently, previous work has shown that deletion of *EMT2* or *tK(CUU)K* did not significantly affect any phenotype evaluated (8). In addition to the CGU anticodon, tK(CUU)K and Emt2 also contain another major identity element for recognition by ThrRS, the G1C71 base pair in the acceptor stem (47), so that these tRNAs are now presumably aminoacylated with threonine instead of lysine or methionine, respectively. The isolation at high temperature of two transformants, KEA17 and KEA24, with a mutation in the anticodon changing lysine or methionine into threonine, respectively, suggests that these mutant tRNAs can take over the function of the unique tRNA^Thr^_CGU_, when it becomes defective or limiting at high temperature. To evaluate this hypothesis, we tested whether the mutant *tK(CUU)K^KEA17^* and *EMT2^KEA24^* can rescue the lethality caused by deletion of *TRT2*. For that purpose, we engineered the tRNA^Lys>Thr^_CUU>CGU_ or the tRNA^Met>Thr^_CAU>CGU_ mutation in a heterozygous *TRT2/trt2*::*NatMX* diploid strain. While the segregants of the parent heterozygous *TRT2/trt2*::*NatMX* diploid strain show the expected 50% viability upon tetrad analysis (Fig. 4E), sporulation of the engineered heterozygous diploid strains now resulted in both cases in 100% viability of the segregants (Fig. 4F and G). A control experiment in which the segregants of the engineered heterozygous *TRT2/trt2*::*NatMX* diploid strains were spotted on YP and 2% glucose medium with nourseothricin confirmed that 50% of the segregants contained the *trt2*::*NatMX* deletion (Fig. 4H and I). Since the *NatMX* marker provides resistance against nourseothricin, segregants without the marker cannot grow. Additional control experiments showed that sporulation of the wild-type strain QD47 (*TRT2*^OEA28^/*TRT2*^ER18alpha^) containing the same genetic background and the same strain engineered with the tRNA^Lys>Thr^_CUU>CGU_ or the tRNA^Met>Thr^_CAU>CGU_ mutation in each case resulted in 100% spore viability (see Fig. S2).

10.1128/mBio.03649-20.2 FIG S2 Tetrad analysis of strain QD47 [OEA28(a)/ER18alpha], QD47 *tK(CUU)K*^KEA17^/*tK(CUU)K*^KEA17^, and *EMT2*^KEA24^/*EMT2*^KEA24^. (A) Tetrad analysis of QD47. (B) Tetrad analysis of QD47 *tK(CUU)K*^KEA17^/*tK(CUU)K*^KEA17^. (C) Tetrad analysis of QD47 *EMT2*^KEA24^/*EMT2*^KEA24^. Download FIG S2, TIF file, 2.9 MB. Copyright © 2021 Deparis et al. 2021 Deparis et al. https://creativecommons.org/licenses/by/4.0/ This content is distributed under the terms of the Creative Commons Attribution 4.0 International license.

### The tRNA^Lys>Thr^_CUU>CGU_ and tRNA^Met>Thr^_CAU>CGU_ anticodon changes do not result in mistranslation.

**FIG 5:**
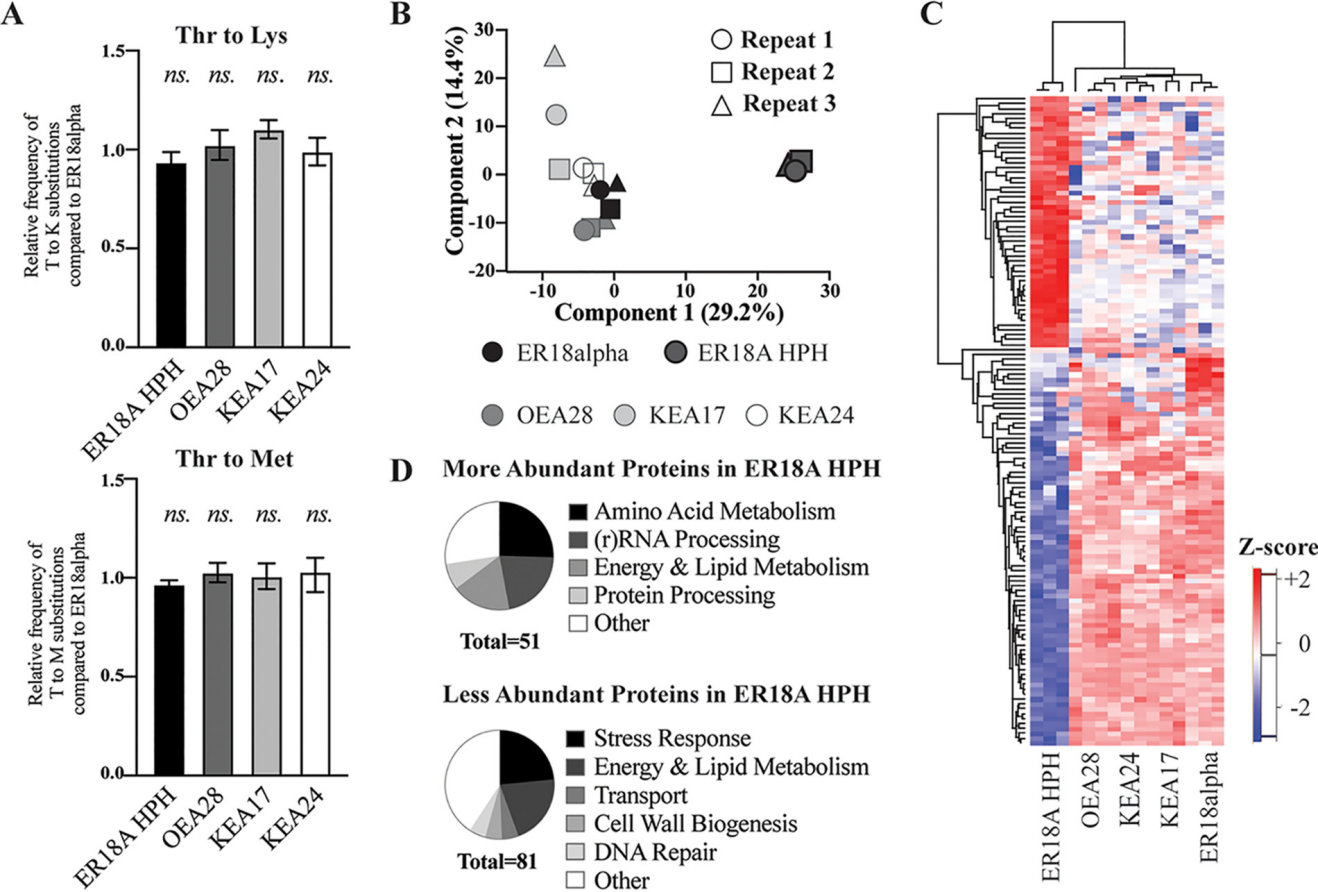
Shotgun proteomics analysis of the host strain, the three WG transformants, and ER18alpha during fermentations at 42°C. (A) Relative frequencies of threonine-to-lysine and threonine-to-methionine amino acid substitutions for ER18A HPH, OEA28 (tRNA*^Thr^_CGU_), KEA17 (tRNA^Lys>Thr^_CUU>CGU_), and KEA24 (tRNA^Met>Thr^_CAU>CGU_) compared to ER18alpha. Error bars indicate the SD between three biological repeats. There is no significant difference between the values measured in any set of four strains (ns, *P* > 0.05; *, *P* < 0.05; **, *P* < 0.01; ***, *P* < 0.001; ****, *P* < 0.0001). (B) Principal-component analysis of all detectable proteins between all strains and their replicates. *x* axis, principal component 1 explaining 29.2% of all variation; *y* axis, principal component 2 explaining 14.4% of all variation. Symbols: circle, repeat 1; square, repeat 2; triangle; repeat 3. (C) Hierarchical clustering of log_2_-transformed z-scores of 132 differentially abundant proteins for five strains (3n; rows). Red indicates higher abundance; blue indicates lower abundance. A secondary clustering (columns) was done to compare differences between strains. (D) GO-term clustering of more-abundant (*n* = 51) and less-abundant (*n* = 81) proteins in ER18A HPH compared to ER18alpha. (For a detailed list of GO terms and associated gene products, see Table S1.)

Despite the fact that tK(CUU)K^KEA17^ and Emt2^KEA24^ can suppress *trt2Δ-*mediated lethality, the remote possibility existed that these tRNAs do no act as direct replacements but that the cells in some way tolerate mistranslation of the threonine encoding ACG codons into lysine or methionine by unknown mechanisms. Mistranslation is known to trigger the stress response (48), which could have a beneficial effect in tolerating high temperature. In addition, the existing knowledge from *in vitro* aminoacylation experiments in Escherichia coli indicated involvement of additional domains in tRNAs besides the anticodon for recognition by their cognate aaRS. Hence, it remained doubtful whether ThrRS could recognize tK(CUU)K^KEA17^ and Emt2^KEA24^ only because of the modification of their anticodon into CGU. To evaluate whether mistranslation happened in the KEA17 (tRNA^Lys>Thr^_CUU>CGU_) and KEA24 (tRNA^Met>Thr^_CAU>CGU_) WG transformants, we performed LC-MS/MS analysis of their whole proteomes. Fermentation experiments were performed at 42°C with the KEA17 (tRNA^Lys>Thr^_CUU>CGU_) and KEA24 (tRNA^Met>Thr^_CAU>CGU_) strains, the parent strain ER18A HPH, the OEA28 (tRNA*^Thr^_CGU_) WG transformant, and the ER18alpha strain, which is isogenic to ER18A but does not contain the destabilizing mutation in *TRT2* and displays regular wild-type heat tolerance. Cell aliquots, containing 2 mg of protein, were harvested after 10 h of fermentation, washed twice with PBS buffer, flash frozen in liquid nitrogen, and stored at −80°C. The samples were subjected to LC-MS/MS analysis of their whole proteomes and analyzed with validated methodology according to Maia et al. (49) (VIB Proteomics Core Facility). Elution spectra after the LC runs indicated appropriate proteome coverage in all samples (see Fig. S3). Next, we quantified peptide counts and compared the number of erroneous threonine-to-lysine and threonine-to-methionine background substitutions in all quantified peptides. This number was very low but was the same in all strains, indicating that there was no significant increase in mistranslation of threonine to lysine or methionine in the KEA17 (tRNA^Lys>Thr^_CUU>CGU_) and KEA24 (tRNA^Met>Thr^_CAU>CGU_) WG transformants (Fig. 5A). We also investigated whether there was a significant reduction in the number of reliably quantifiable Thr-containing peptides (*n* = 3,663 on a total of *n* = 18,059 peptides). However, when we plotted the distribution of corrected log_2_-fold change for all peptides and for Thr-containing peptides separately, the two plots clearly showed complete overlap (see Fig. S4). This indicated that there was no significant reduction in the number of Thr-containing peptides in the KEA17 (tRNA^Lys>Thr^_CUU>CGU_) and KEA24 (tRNA^Met>Thr^_CAU>CGU_) WG transformants, again contradicting any significant mistranslation of threonine encoding ACG codons into lysine or methionine.

10.1128/mBio.03649-20.3 FIG S3 LC chromatograms for quantification of total protein amounts. (A) ER18alpha. (B) ER18A HPH. (C) OEA28. (D) KEA17. (E) KEA24. Three biological repeats per strain were analyzed. Download FIG S3, TIF file, 2.6 MB. Copyright © 2021 Deparis et al. 2021 Deparis et al. https://creativecommons.org/licenses/by/4.0/ This content is distributed under the terms of the Creative Commons Attribution 4.0 International license.

10.1128/mBio.03649-20.4 FIG S4 Detection of potential misincorporation of lysine or methionine residues instead of threonine in ER18A HPH and the three transformants relative to the reference strain. Distribution of corrected log_2_-fold changes for all peptides (red) and for Thr-containing peptides only (blue). If the levels of Thr-containing peptides would be consistently lower (expected in the case of reduced Thr incorporation), their distribution should appear shifted to the left. The graphs are a combination of three technical repeats per strain. Download FIG S4, TIF file, 2.6 MB. Copyright © 2021 Deparis et al. 2021 Deparis et al. https://creativecommons.org/licenses/by/4.0/ This content is distributed under the terms of the Creative Commons Attribution 4.0 International license.

10.1128/mBio.03649-20.6 TABLE S1 GO terms and associated gene products. Download Table S1, DOCX file, 0.03 MB. Copyright © 2021 Deparis et al. 2021 Deparis et al. https://creativecommons.org/licenses/by/4.0/ This content is distributed under the terms of the Creative Commons Attribution 4.0 International license.

### The three WG transformants recovered the wild-type protein abundance profile.

We next analyzed whether there was any differential protein abundance in cells fermenting at 42°C of the WG transformants compared to the parent strain ER18A HPH and control strain ER18alpha, which might have been caused by the modified tRNA utilization profile. Strikingly, among the 2,047 quantifiable proteins 132 proteins showed a different abundance in the ER18A HPH strain compared to the control strain ER18alpha, as well as to the other three strains (Fig. 5B and C). This indicates that the destabilizing mutation in *TRT2* and thus the resulting defect in the tRNA^Thr^_CGU_ affects the synthesis of a significant subset of proteins at 42°C, with 81 proteins being reduced in expression level and 51 proteins of which the expression level has increased. The three WG transformants did not show any significant deviation in protein abundance at 42°C compared to the control strain ER18alpha, indicating that all three suppressor mutations completely abolished the detrimental effect of the defective tRNA^Thr^_CGU_ on the proteome expression profile. The proteins upregulated or downregulated at 42°C in the ER18A HPH strain belonged to different GO terms (Fig. 5D). For a detailed list of GO terms and associated gene products, see Table S1 in the supplemental material. Interestingly, the largest category of downregulated proteins was involved in stress response, while we have previously shown that there is a relative increase in the number of ACG-encoded threonine residues in heat stress-induced proteins (Fig. 3E). This inappropriate heat stress response may have exacerbated the fermentation defect at 42°C in the ER18A HPH strain.

### Transcriptional expression levels of tRNAs and tRNA degradation enzymes in the WG transformants.

**FIG 6:**
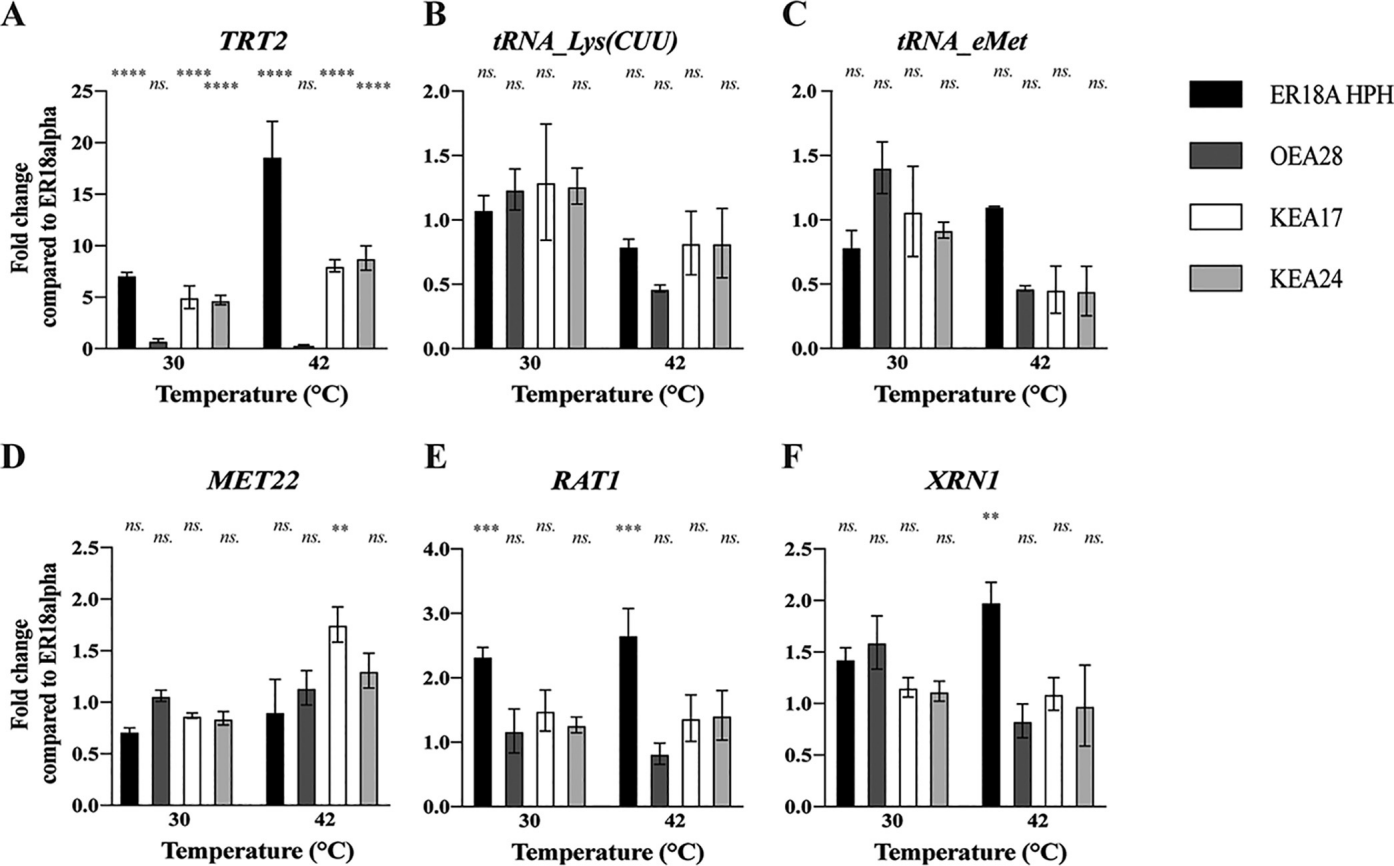
Expression analysis of the three tRNAs and selected RTD pathway genes in the host strain, ER18A HPH, the three WG transformants [OEA28 (tRNA*^Thr^_CGU_), KEA17 (tRNA^Lys>Thr^_CUU>CGU_), and KEA24 (tRNA^Met>Thr^_CAU>CGU_)], and ER18alpha during fermentation at 30 and 42°C. The fold change in expression level was measured by RT-qPCR during fermentation at 30 and 42°C compared to ER18alpha of *TRT2* and the two related tRNA^Thr^s (A), tRNA^Lys^ (*n* = 14) (B), and tRNA^eMet^ (*n* = 5) (C). RT-qPCR analysis of selected genes involved in the rapid tRNA decay (RTD) pathway. (D) *MET22*, encoding a regulator; (E) *RAT1*, encoding an RNase; (F) *XRN1*, encoding an RNase. Error bars represent the SD of three biological repeats with each of three technical repeats (ns, *P* > 0.05; *, *P* < 0.05; **, *P* < 0.01; ***, *P* < 0.001; ****, *P* < 0.0001). *TDH2* and *FBA1* were used as reference genes.

We next performed RT-qPCR for determination of the transcriptional expression level of the *TRT2*, *tRNA_Lys(CUU)*, and *tRNA_eMet* genes in the WG transformants at 30 and 42°C in comparison to the parent strain ER18A HPH and the control strain ER18alpha. For this purpose, cells were harvested after 8 and 10 h of fermentation at 30 and 42°C, respectively. The results are expressed as fold change compared to the transcriptional expression level in the ER18alpha strain (Fig. 6). The expression level of *TRT2* was the same in the OEA28(tRNA*^Thr^_CGU_) WG transformant and the control strain ER18alpha at 30°C, indicating that the modified base pair in the anticodon stem did not affect *TRT2* expression and thus likely also not the functionality of the Trt2 tRNA^Thr^_CGU_ (Fig. 6A). On the other hand, the KEA17 (tRNA^Lys>Thr^_CUU>CGU_) and KEA24 (tRNA^Met>Thr^_CAU>CGU_) WG transformants, and even more pronounced the ER18A HPH strain, showed a clearly elevated *TRT2* expression level at 42°C and already to a lower extent at 30°C. This suggests that the cells tried to overcome the deficiency in the *TRT2*-encoded tRNA^Thr^_CGU_ by increasing the *TRT2* expression level. The expression level of the *tRNA_Lys(CUU)* and *tRNA_eMet* genes was not or not much affected at 30°C, whereas at 42°C there was a somewhat reduced expression with at most 50% in some of the strains (Fig. 6B and C). Also, expression of the genes involved in the RTD pathway for tRNA breakdown was not much affected. The most conspicuous change was the increase in *RAT1* and *XRN1* expression at 42°C in the ER18A HPH strain compared to the control strain (Fig. 6E and F), which may be due to the strong upregulation of *TRT2* expression under this condition (Fig. 6A).

### The UPR pathway is induced at 42°C in the ER18A HPH parent strain but not in the WG transformants.

**FIG 7:**
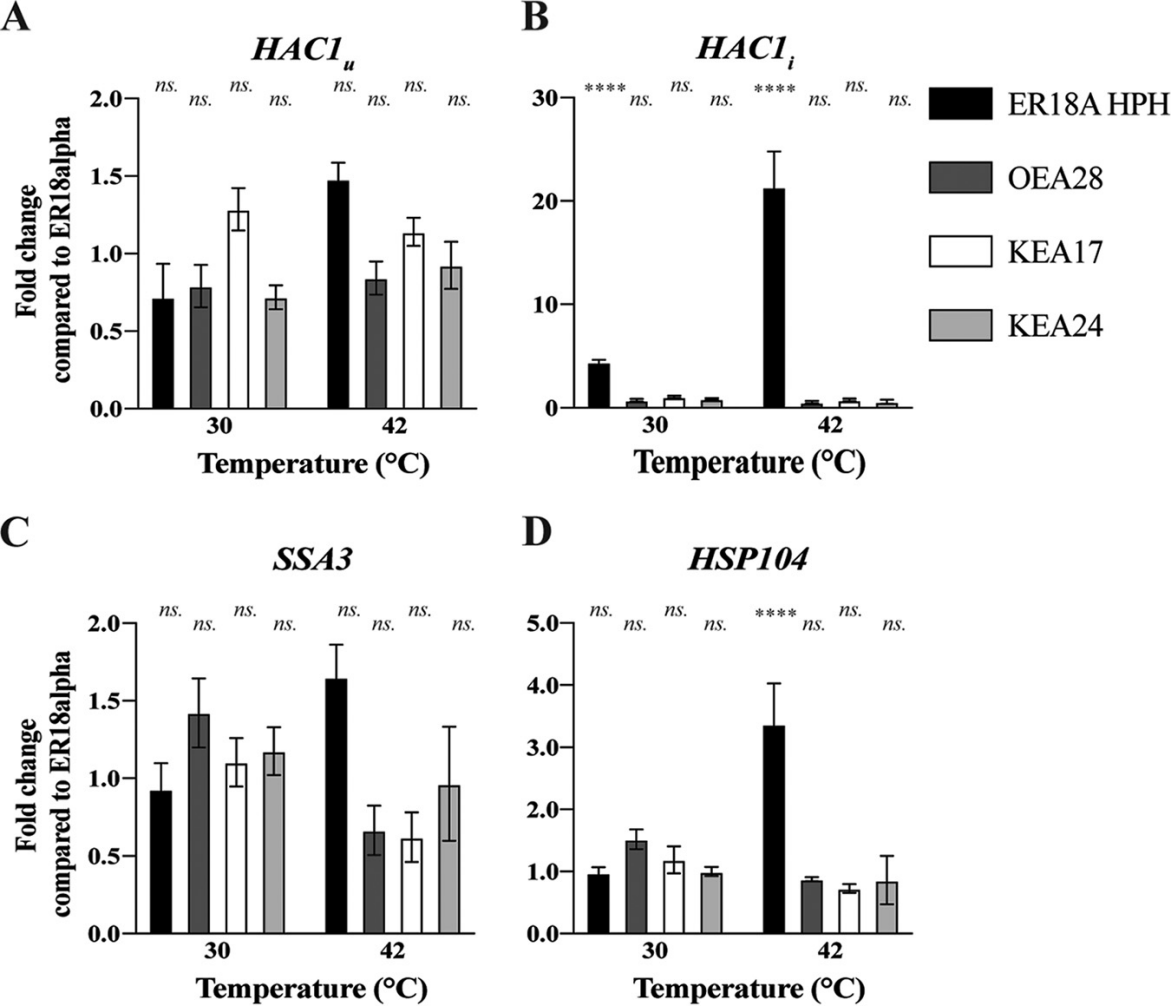
Expression analysis of UPR and target genes in the host strain, ER18A HPH, the three WG transformants [OEA28 (tRNA*^Thr^_CGU_), KEA17 (tRNA^Lys>Thr^_CUU>CGU_), and KEA24 (tRNA^Met>Thr^_CAU>CGU_)], and ER18alpha during fermentation at 30 and 42°C. The fold change in expression level was measured by RT-qPCR during fermentation at 30 and 42°C compared to ER18alpha of genes involved in the unfolded protein response (UPR) pathway. (A to D) Nonspliced long transcript (inactive) *HAC1* (=*HAC1_u_*) (A), Spliced short transcript (active) *HAC1* (*HAC1_i_*_)_ (B), and two target genes encoding heat shock proteins involved in protein folding: *SSA3* (C) and *HSP104* (D). Error bars represent the SD of three biological repeats with each of three technical repeats (ns, *P* > 0.05; *, *P* < 0.05; **, *P* < 0.01; ***, *P* < 0.001; ****, *P* < 0.0001). *TDH2* and *FBA1* were used as reference genes.

We also measured induction of the UPR pathway under the same conditions. This pathway is activated when misfolded proteins accumulate due to certain stresses (e.g., temperature stress). The pathway targets the Hac1 transcription factor that supports induction of multiple genes encoding stress-protective ER proteins. *HAC1* transcripts are only spliced to generate an active mRNA when misfolded protein aggregates are formed. We quantified the mRNA expression levels of the spliced and non-spliced variants of *HAC1* mRNA for which ER18A HPH showed a 20-fold increase in the level of spliced *HAC1* mRNA compared to the level in the control strain ER18alpha (Fig. 7A and B). On the other hand, all three WG transformants—OEA28 (tRNA*^Thr^_CGU_), KEA17 (tRNA^Lys>Thr^_CUU>CGU_), and KEA24 (tRNA^Met>Thr^_CAU>CGU_)—did not show any evidence for significant induction of the UPR pathway compared to the control strain ER18alpha (Fig. 7A and B). This again confirmed that the WG transformants behaved like a wild-type strain at 42°C. While transcription of the chaperone Ssa3 was not significantly induced compared to the control strain, *HSP104*, which encodes a cytoplasmic chaperone and functions as effector of the UPR pathway, showed significantly higher transcriptional activation at 42°C compared to the control strain ER18alpha (Fig. 7C and D). On the other hand, none of the three WG transformants showed any induction of the two target genes at 42°C and therefore showed a behavior similar to that of the control wild-type strain ER18alpha (Fig. 7C and D).

### Sequence analysis of additional whole-genome transformants.

We assessed whether the generation of tRNA anticodon mutations also happened in other WG transformants that we isolated independently under heat stress. First, we screened the remaining transformants for the presence of the two previously identified anticodon mutations and the stabilizing mutation in *TRT2*, which revealed that KEA21 acquired the same CUU-to-CGU anticodon mutation as KEA17 in *tK(CUU)K*. Seven strains that did not acquire a mutation in these genes, of which five with a similarly improved thermotolerance as KEA17, KEA24, and OEA28 and two with a smaller improvement, were then submitted to whole-genome sequence analysis (Fig. 1B, gray lines with different symbols).

We identified tRNA anticodon mutations in six out of the seven transformants (see Table S2). One transformant acquired a mutation in *tT(AGU)H*, encoding tRNA^Thr^_AGU_, in which the AGU anticodon was mutated to CGU. In five other transformants, *EMT5*, also encoding a tRNA^eMet^, acquired an A>G mutation in the anticodon, similar to the *EMT2* anticodon mutation in KEA24. Only one transformant (OEA57) did not contain an anticodon mutation, but it also showed a lower level of thermotolerance as the transformants with an anticodon mutation. These WG transformants also contained between 1 and 15 other SNPs.

10.1128/mBio.03649-20.7 TABLE S2 Anticodon mutations and number of other SNPs in the genome of additional heat-tolerant WG transformants. Download Table S2, DOCX file, 0.03 MB. Copyright © 2021 Deparis et al. 2021 Deparis et al. https://creativecommons.org/licenses/by/4.0/ This content is distributed under the terms of the Creative Commons Attribution 4.0 International license.

### Apparent anticodon switches during evolution.

**FIG 8:**
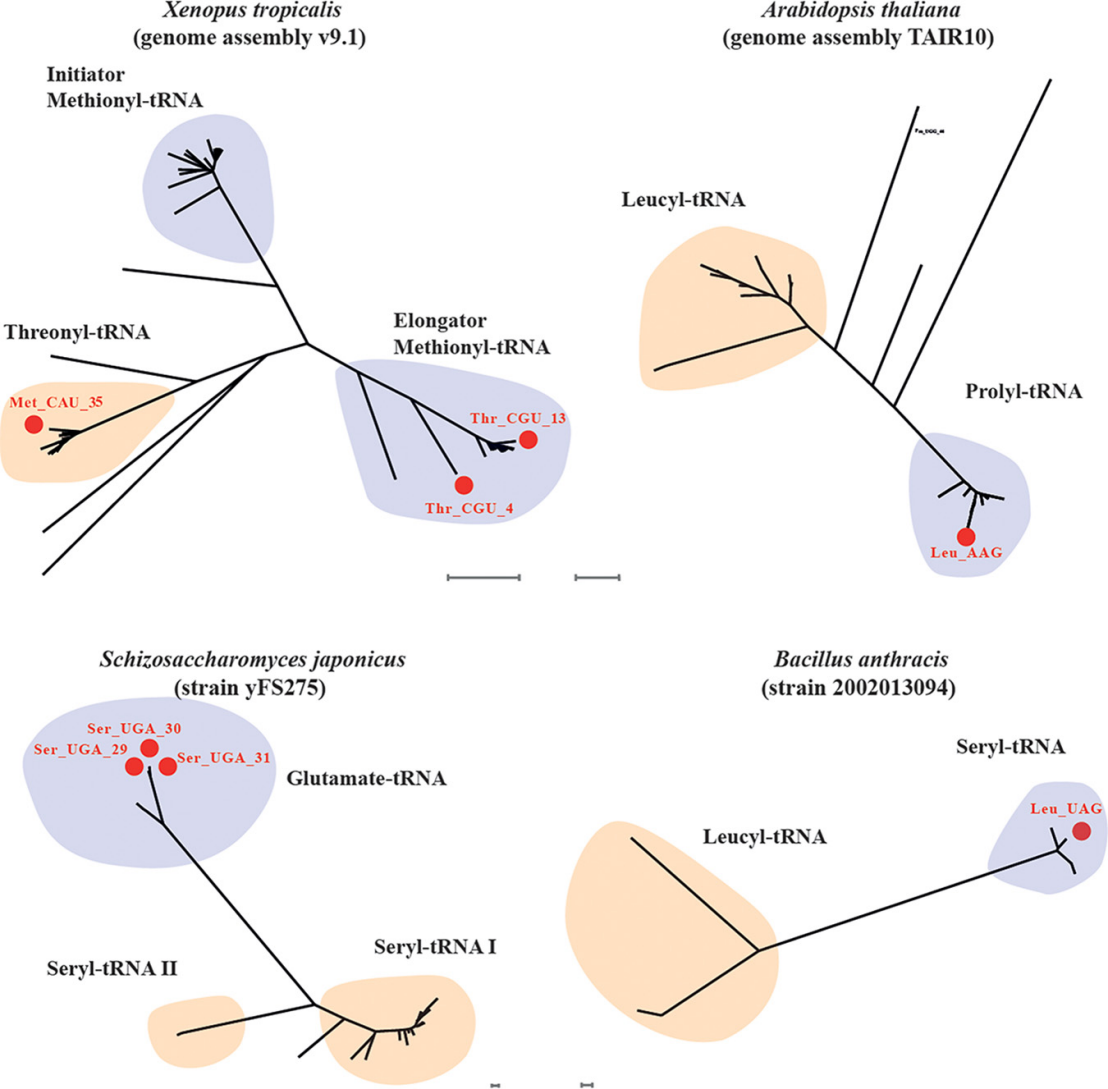
Dendrogram of aligned tRNA sequences from four species showing amino acid-based clades containing one, two, or three tRNAs specifying a different amino acid. The same methionine-to-threonine switched tRNA as that found in the whole-genome transformant KEA24 (tRNA^Met>Thr^_CAU>CGU_) is present two times in the clade of methionyl-tRNAs in *Xenopus tropicalis* (v9.1; scale bars, 0.1), Arabidopsis thaliana (TAIR10; scale bars, 0.1), *Schizosaccharomyces japonicus* strain yFS275 (scale bars, 0.01), and Bacillus anthracis strain 2002013094 (scale bars, 0.01).

We have evaluated whether the same or similar anticodon switches might have occurred during evolution by checking phylogenetic trees of tRNA genes in yeast and in other organisms (70 archaeal, 576 bacterial, and 495 eukaryotic strains/species) for the presence of an aberrant anticodon in the encoded tRNA. For yeast specifically, we have used the 1,011 available genome sequences (50) and found several examples of switched anticodons in tRNA encoding genes (Table 2). These were all different from those present in the WG transformants KEA17 (tRNA^Lys>Thr^_CUU>CGU_) and KEA24 (tRNA^Met>Thr^_CAU>CGU_). Also, in phylogenetic trees of tRNA genes in bacteria, fungi, plants and animals, we found evidence for this type of anticodon switching (Fig. 8). Strikingly, we found exactly the same anticodon switch tRNA^Met>Thr^_CAU>CGU_, as we found in the KEA24 (tRNA^Met>Thr^_CAU>CGU_) WG transformant, in a tRNA^Met^_CAU_ gene family in *Xenopus tropicalis* (Fig. 8) and in six other species, Danio rerio, *Gasterosteus aculeatus*, *Gadus morhua*, *Stronglycentrotus purpuratus*, *Allomyces macrogynus*, and *Cronartium comandrae*. Also, the reverse switch, from tRNA^Thr^_CGU_ to tRNA^Met^_CAU_, was present in *X. tropicalis* (Fig. 8). Similar anticodon switches were present in phylogenetic tRNA gene families in Arabidopsis thaliana (Fig. 8), *Schizosaccharomyces japonicus* (Fig. 8), and Bacillus anthracis (Fig. 8). Hence, this type of anticodon switching mutations in tRNA genes seems to have occurred throughout evolution.

**TABLE 2:** Presence of anticodon mutations that cause amino acid changes in S. cerevisiae strains^*a*^

Original tRNA	Original amino acid	Anticodon-mutated tRNA	New amino acid	S. cerevisiae strain(s) with anticodon-mutated tRNA gene(s) in their genome(s)
*tA*(*AGC*)*J*	Alanine	*tR(UGC)J*	Arginine	ARN
*tA*(*AGC*)*M1*	Alanine	*tR(CGC)M1*	Arginine	AEB, AQT, ASH, BBG, BRP, BRQ, BSI, CBR, CFC, CFG, CFH
*tC*(*GCA*)*P2*	Cysteine	*tS(CCA)P2*	Serine	BHM
*tM*(*CAU*)*D*	Methionine	*tI(UAU)D*	Isoleucine	CFD, CFM
*tP*(*AGG*)*C*	Proline	*tT(ACU)C*	Threonine	CBR, CFG, CFH
*tR*(*ACG*)*K*	Arginine	*tH(AUG)K*	Histidine	ALN, ALP, ALV, AMN, APL, BBC, CFL, CFN, CFP
*tR*(*UGU*)*B*	Arginine	*tK(UUU)B*	Lysine	ARI, BEK, BEM
*tR*(*UGU*)*G1*	Arginine	*tK(UUU)G1*	Lysine	ARI
*tV*(*AAC*)*E2*	Valine	*tL(AAG)E2*	Leucine	CRE
*tV*(*AAC*)*M1*	Valine	*tI(AAU)M1*	Isoleucine	AEC
*tV*(*UAC*)*D*	Valine	*tG(UCC)D*	Glycine	BGM

a The S. cerevisiae genomes sequences screened are those of the 1,011 genome project (50).

## DISCUSSION

The isolation of strains displaying a superior, selectable phenotype after transformation with the whole gDNA from another organism has been reported previously. However, the underlying mechanism has only been documented clearly in the case of bacterial WG transformants selected on the basis of antibiotic resistance (32–35). In this case, SNPs in resistance-conferring genes from the donor strain have been transferred in various combinations to the host strain by homologous recombination allowing identification of the causative mutation(s). On the other hand, the few reports on WGT with eukaryotes apparently resulted in integration of large fragments of donor gDNA into the host strain gDNA (37, 38). This is what we also observed upon selection for an antibiotic resistance marker, HPH, present in the donor gDNA and selected for in the host strain transformants. To gain hygromycin B resistance, a complete functional HPH gene is required. Hence, after WGT of S. cerevisiae with gDNA from the thermotolerant species K. marxianus or *O. polymorpha* and selection for higher thermotolerance, we expected to find in the genome of the transformants one or more large fragments of heterologous gDNA containing one or more causative genetic elements. Instead, we found a surprisingly low number of SNPs, which, in addition, were not even present in the gDNA of the donor species. The further identification of the causative SNPs being mutations in the stem or anticodon of tRNAs, apparently rescuing the instability of the mutant Trt2^Thr^_CGU_ tRNA in the ER18A host strain at higher temperature, provides a logical explanation for the mechanism conferring higher thermotolerance in the WG transformants. However, it does not explain how these rescuing mutations have been generated.

**FIG 9:**
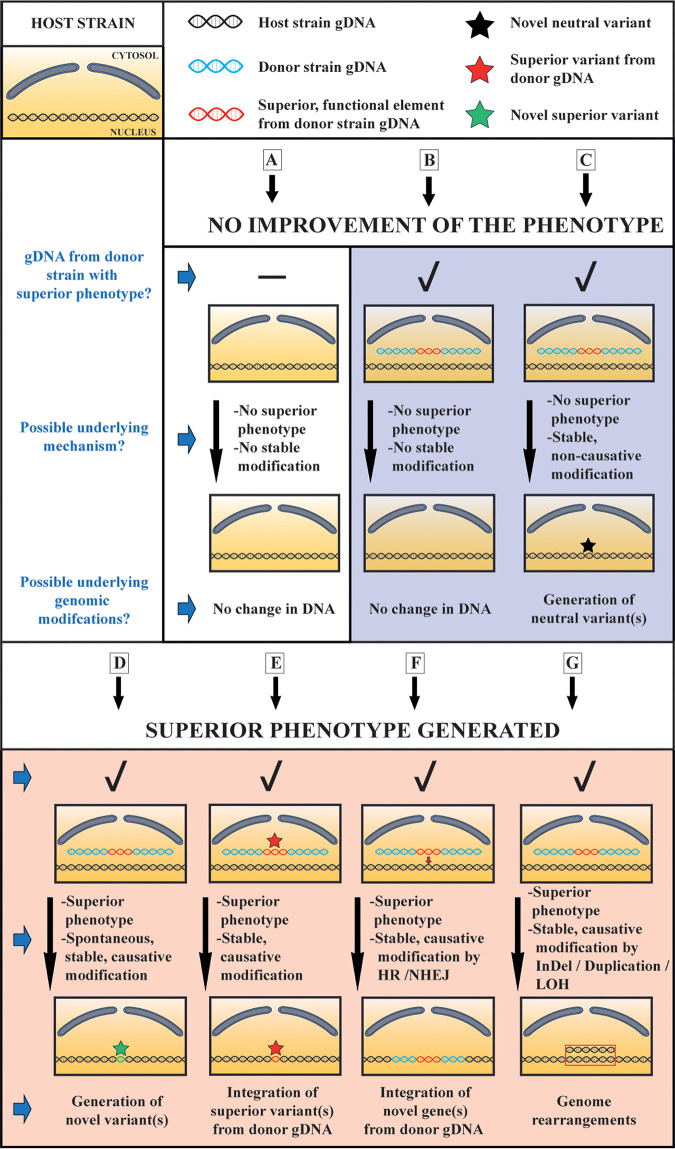
Schematic overview of possible scenarios for generation of mutant strains with a superior phenotype during WGT with gDNA from a superior donor strain. WGT with gDNA from a superior donor strain (blue DNA strand), possibly containing a genetic element able to confer the superior phenotype for which selection is applied (red DNA fragment), could in principle result in multiple outcomes for the gDNA in the host strain (black DNA strand). When transformation is performed, as shown for scenario A, without any gDNA (water control) or with random DNA from organisms lacking the superior trait, no transformants are obtained, at least under the conditions and time span applied in our experiments. When a gDNA fragment from the superior donor strain, containing a genetic element able to confer the superior phenotype, is taken up by the host cell but the latter does not generate any modification in its gDNA (scenario B) nor any modification able to confer the selectable phenotype (scenario C, black star) before the donor gDNA fragment has been lost during cell proliferation, no superior transformants are obtained. When the host strain has been able to acquire a novel mutation (SNP) in its gDNA (scenario D, green star) or has been able to incorporate a mutation (SNP) from the donor gDNA (scenario E, red star) that confers the selectable phenotype before the donor gDNA fragment has been lost during cell proliferation, a viable superior and stable transformant will have been generated. When the host strain has been able to incorporate, by homologous recombination (HR) or nonhomologous end joining (NHEJ), a complete functional element (e.g., containing a full gene) from the donor gDNA that confers the selectable phenotype before the (remainder of the) donor gDNA fragment has been lost during cell proliferation, a viable superior and stable transformant will have been generated (scenario F). Finally, when the host strain has been able to generate any other genome modifications, such as large-scale chromosome duplications, indels, and/or loss of heterozygosity (LOH) that confers the selectable phenotype, before the donor gDNA fragment has been lost during cell proliferation, a viable superior and stable transformant will have been generated (scenario G).

We depict in Fig. 9 a number of possible scenarios. First of all, when the transformation was performed with water or with random DNA derived from nonthermotolerant species, including the gDNA from the host strain, no stable thermotolerant strains were obtained, contradicting the occurrence of spontaneous mutations in the host strain gDNA (Fig. 9A). This observation also contradicts that foreign DNA, or any DNA, would act as a general mutagen triggering many random mutations in the genome, after which strains with mutations conferring higher thermotolerance would be selected. The inability to isolate thermotolerant transformants with gDNA from nonthermotolerant species also appears to contradict that the wild-type *TRT2* gene in the donor gDNA protected the host strain until a spontaneous mutation occurred that could complement its defective *trt2*^ER18A^ gene. In all the subsequent scenarios, a fragment of the donor gDNA, containing a genetic element that confers higher thermotolerance, is taken up by the host cell without being stably integrated into the host gDNA (scenarios B, C, D, and G) or with stable integration of at least part of the heterologous DNA conferring higher thermotolerance into the host DNA (scenarios E and F). Expression of the foreign thermotolerance conferring genetic element, allows the yeast to survive and proliferate for a certain time period at the higher temperature before this fragment is lost again. When the yeast fails to generate any SNPs (scenario B) or only generates neutral SNPs or any other type of genomic modification not conferring higher thermotolerance (scenario C), during this time period, no viable transformants will be obtained. On the other hand, when in the host strain during this time period a spontaneous SNP occurs conferring higher thermotolerance (scenario D), or integrates by homologous recombination a SNP from the donor gDNA conferring higher thermotolerance (scenario E) or integrates a larger fragment of the donor gDNA containing a functional genetic element conferring higher thermotolerance (scenario F), or undergoes any other type of genomic modification (rearrangement, change in ploidy, etc.) conferring higher thermotolerance (scenario G), viable and stable transformants will be obtained. They will have lost the free original foreign gDNA fragment (linear or circular), which they no longer need to remain thermotolerant. The bacterial WG transformants isolated based on antibiotic resistance (32–35) were apparently generated by scenario E, while the eukaryotic WG transformants in yeast and rice (37, 38) appear to have been generated by scenario F and/or G. In our case, the thermotolerant WG transformants appear to have been generated by scenario D. Although we do not have direct evidence in this respect, the inability to isolate WG transformants at higher temperatures or with gDNA from non-thermotolerant strains, is consistent with this mechanism. For all mechanisms in which (a fragment of) the donor gDNA only plays a protective role during spontaneous occurrence of selectable mutations in the host gDNA, it appears likely that isolation of WG transformants has a higher chance of success with gDNA from more closely related species compared to distantly related species, since proper expression of one or more protective gene products at least for a certain period of time is required.

If the proposed mechanism D is indeed correct, it raises interesting questions with respect to the GMO (Genetically Modified Organism) status of the transformants. In that case, the strains are transiently transgenic and permanently cisgenic. WGT and the occurrence of spontaneous mutations are natural processes that should not make the resulting strains per definition GMO’s. On the other hand, the use of heterologous DNA may raise suspicion and create a dilemma for application of current GMO regulations in many countries (51, 52).

Whole-genome sequence analysis revealed a very low number of nonsynonymous SNPs in the genome of the three selected WG transformants with enhanced thermotolerance of which in each case a tRNA mutation was shown by allele exchange to be causative. This led to the finding that the ER18A strain contained a mutation in the anticodon stem of *TRT2*-encoded tRNA^Thr^_CGU_ causing loss of base pairing, apparently reducing the structural stability of the tRNA^Thr^_CGU_, which is exacerbated at higher temperature. At 30 and 35°C, no difference in fermentation performance was observed, while it was clearly compromised at 42°C. Expression analysis of *TRT2*, on the other hand, revealed an ∼7-fold upregulation at 30°C and an ∼20-fold upregulation at 42°C, indicating that also at 30°C the aberrant tRNA^Thr^_CGU_ caused a cellular response. All three causative tRNA mutations in the WG transformants apparently rescued the temperature sensitivity of the ER18A mutant tRNA^Thr^_CGU_, either by a mutation in the OEA28 tRNA^Thr^_CGU_ resulting in noncognate, but proper base pairing in the anticodon stem, or by mutations in the anticodon of a lysine and methionine tRNA providing an alternative source of tRNA^Thr^_CGU_. The latter situation has been reported previously when the unique *HXS1* gene encoding tRNA^Arg^_CCU_ in yeast had been deleted causing a slow-growth phenotype and the resulting strain submitted to evolutionary adaptation (29). It generated suppressor mutations in isoacceptor tRNA encoding genes, providing an alternative source of tRNA^Arg^_CCU_ and restoring the normal growth rate.

The question can be raised why the subtle base pairing defect in tRNA^Thr^_CGU_ was so selective that it led to different suppressor mutations all overcoming the clear growth and fermentation defect of the strain in other ways. There seem to be several arguments. First, in the genome there is only one gene, *TRT2*, encoding tRNA^Thr^_CGU_. Hence, its malfunctioning cannot be complemented via noncanonical or wobble base pairing by one of the other two threonine tRNA species (8). Second, the most highly expressed genes upon temperature upshift to 37°C contain a higher proportion of codons matching the CGU anticodon than the average expressed gene pool. Finally, these genes, which require a higher proportion of tRNA^Thr^_CGU_ for their translation, are all significantly induced upon temperature upshift, further amplifying the requirement for tRNA^Thr^_CGU_ (46). This is in agreement with the observation that stress-responsive genes contain a higher-than-average percentage of rare codons (25, 53).

The precise mechanism causing the thermosensitivity of Trt2^ER18A^ is not clear. Since the anticodon stem is important for interaction with ThrRS (54), the tRNA possibly cannot be aminoacylated properly by ThrRS at high temperature. The importance of the anticodon stem for recognition of the cognate tRNA by its aaRS has been shown for valine-tRNA synthetase, *Ec*ValRS, which has a 50-fold-decreased affinity for tRNA^Val^ when the U29A41 base pair (identical position as the G28⋅C40 base pair in tRNA^Thr^_CGU_) was exchanged for a noncognate C29G41 base pair (55, 56). Alternatively, or in addition, the modified anticodon stem may compromise anticodon-codon or tRNA-ribosome interaction at 42°C, for which examples have previously been reported (57). In both cases, mistranslation and alteration of the translatome profile may be the result, as we have observed with the LC-MS/MS analysis (Fig. 5B and C).

In our work, tetrad analysis has shown that the lethality caused by *trt2Δ* can apparently not be overcome by noncanonical UG base pairing or inosine wobbling at the third position of the codon, as has also been observed in previous research (8). On the other hand, the presence of the tRNA^Lys^_CUU>CGU_ or the tRNA^eMet^_CAU>CGU_ completely suppressed *trt2Δ* lethality (Fig. 4). Low levels of mistranslation can be tolerable for cell growth (58, 59), and such levels could be below the detection level of our proteomic mass spectrometry analysis. However, previous work has shown that even low levels of mistranslation can have an effect on transcription of the targets that we have tested (*HAC1* and the heat shock protein genes), as well as on protein abundance (48), neither of which we were able to detect. In our case mistranslation of all Thr^ACG^ codons into Lys or Met would theoretically result for both in 24,222 mutations in the genome. Even with a low level of mistranslation, there would still be more than 20,000 mutations in the genome, which is unlikely to be compatible with regular cell growth and viability. In addition, 366 Thr^ACG^ codons have been identified as putative phosphorylation sites (see Materials and Methods), which would all disappear upon mutagenesis into Lys or Met. Hence, we considered it highly unlikely that all or even a significant part of the Thr^ACG^ codons would be mistranslated into Lys or Met. To obtain definite proof for the absence of significant mistranslation, we have performed LC-MS/MS analysis of the whole proteome. It clearly indicated that the number of Lys or Met containing peptides was not significantly increased and the number of Thr containing peptides not significantly reduced. Previous work has shown that significant mistranslation can be detected in S. cerevisiae using proteomic mass spectrometry (60). Hence, we are bound to conclude that the ThrRS can now recognize the anticodon-mutated tK(CUU)K or the anticodon-mutated Emt2 for aminoacylation with Thr and that the lysyl-tRNA synthetase (LysRS) and methionyl-tRNA synthetase (MetRS) can apparently no longer recognize, at least not to a great extent, their cognate tRNA solely due to the anticodon mutation. The RT-qPCR results show that in the WG transformants the UPR is not induced at 42°C, which is consistent with the absence of significant misfolding of proteins. The latter would likely occur if significant mistranslation would take place. In spite of this, the question can be raised whether our results are not in contradiction with the many reports documenting the importance of other domains in the tRNAs that are important for recognition by aaRSs. In E. coli, it has been shown that the main discriminator bases of tRNAs for recognition by *Ec*ThrRS are the anticodon and the G1C72 and C2G71 base pairs, which correspond to G1C71 and C2G70 in *Sc*Trt2 (54, 61). Furthermore, it was shown that, unlike for several other aaRSs, the terminal acceptor base, A73, is not essential for proper aminoacylation by *Ec*ThrRS (61). Our results, however, do not contradict the existence of other ThrRS recognition domains in tRNA^Lys^_CUU>CGU_ or tRNA^eMet^_CAU>CGU_, since these domains are identical in tRNA^Thr^_CGU_ (see Fig. S5). This explains why the sole change in the anticodon is enough for ThrRS to recognize the two mutant tRNAs. Hence, our results do not imply that the anticodon is a sufficient identity determinant for recognition by ThrRS. In E. coli, it has been shown that changing the anticodon in tRNA^Met^_CAU_ caused an increase in affinity of *Ec*ThrRS with 4 orders of magnitude, while simultaneously decreasing affinity of *Ec*MetRS by >5 orders of magnitude (62). Our results are also consistent with previously reported data that the acceptor base is likely not involved in substrate recognition by EcThrRS (54), since tRNA^Thr^_CGU_ and tRNA^Lys^_CUU_ have a different acceptor base (A versus U). There are many more codons that can mutate into the threonine AGU codon matching the CGU anticodon. Preferential isolation of mutants in selected tRNA genes to overcome the defective tRNA^Thr^_CGU_ could therefore be explained by requirement for the other ThrRS recognition domains to be identical to those in tRNA^Thr^_CGU_. In addition, other domains in certain tRNAs may prevent ThrRS from recognizing the alternative tRNA as a proper substrate (55).

10.1128/mBio.03649-20.5 FIG S5 Structures of native and adapted yeast tRNA^Thr^. Threonyl tRNA-synthetase is known to aminoacylate tRNA^Thr^_CGU_ (A), tRNA^Thr^_UGU_ (B), and tRNA^Thr^_AGU_ (C). In addition, we provide evidence that it can aminoacylate tRNA^Lys^_CUU>CGU_ (D) and tRNA^eMet^_CAU>CGU_ (E and F), when the original anticodon is mutated to the threonine anticodon. The known positive determinants are circled in red (47). The residues that are common in all tRNAs are indicated in orange, and additional residues common in threonine tRNAs are indicated in blue. Download FIG S5, TIF file, 2.6 MB. Copyright © 2021 Deparis et al. 2021 Deparis et al. https://creativecommons.org/licenses/by/4.0/ This content is distributed under the terms of the Creative Commons Attribution 4.0 International license.

Our work reveals that compromised functionality of specific tRNAs under certain stress conditions, may facilitate the selection of mutants in the anticodons of other tRNAs providing an alternative source of functional tRNA to replace or support the defective tRNA. The frequent selection of tRNA suppressor mutations may be facilitated by the increased mutagenesis rates of the tRNA genes during cell proliferation, which is due to transcription-induced mutagenesis (30). Although the tRNA^Thr^_CGU_ in the ER18A strain was not defective at regular temperatures, or at least not to such an extent that it significantly compromised fermentation, its reduced functionality at higher temperature was apparently enough to serve as driving force for the selection of anticodon mutations in other tRNAs. Hence, as opposed to the artificial growth deficiency under regular conditions established by the deletion of yeast tRNA^Arg^_CCU_ in the work of Yona et al. (29), in our case the subtle mutation naturally present in tRNA^Thr^_CGU_ compromised functionality of the tRNA specifically under a high-temperature stress condition. Our own phylogenetic analysis, covering a more extensive range especially for eukaryotic species, confirms that such anticodon switching mutations have been selected and maintained multiple times in evolution, including exactly the same mutation in the anticodon of tRNA^Met^_CAU_ in *X. tropicalis* and six other species, which all generated an alternative tRNA^Thr^_CGU_, as we found in our work. Hence, compromised tRNA functionality may occur quite frequently under specific stress conditions in nature, and therefore selection under stress may have been the driving force for the frequent occurrence of these tRNA anticodon mutations in evolution (63). Interestingly, the *trt2*^ER18A^ mutation has recently been identified as a causative mutation in QTL analysis of very high acetic acid tolerance with the ER18A strain as one of the parent strains (64). This indicates that tRNA mutations affect tolerance to multiple types of stress.

In the phylogenetic analysis of the tRNA genes it is sometimes difficult to judge whether a genuine anticodon mutation has occurred because of low sequence similarity in general in the tRNA gene families. As a result, the occurrence of anticodon mutations might be overestimated, especially in archaeal, bacterial, and lower eukaryotic genomes (29, 31). The apparent presence of anticodon mutations in tRNA gene families has been noted previously in sequenced genomes of many organisms throughout evolution (29). However, the functionality of the mutant tRNAs had not been investigated. Here, we showed that the anticodon mutant tRNAs, tRNA^Lys>Thr^_CUU>CGU_ and tRNA^Met>Thr^_CAU>CGU_, can take over the function of the *TRT2*-encoded tRNA^Thr^_CGU_ in the *trt2Δ* mutant. This makes it highly likely that the same mutant tRNA^Met>Thr^_CAU>CGU_ present in *X. tropicalis* and six other species, as we found in yeast, is also functional.

In summary, we have demonstrated that WGT is a powerful technology for improvement of selectable traits in yeast cell factories. A major advantage appears to be the very low number of mutations introduced in the genome. On the one hand, this greatly facilitates identification of the causative mutation and, on the other hand, it minimizes the risk for detrimental side effects on other traits of industrial importance. Furthermore, we have shown that anticodon mutations in other tRNAs are enough to switch recognition between aaRSs and to take over in this way the function of a defective tRNA under specific stress conditions. This may explain the selection of anticodon mutants in evolution and thus their frequent occurrence in phylogenetic tRNA families.

## MATERIALS AND METHODS

### Yeast strains and media.

Yeast strains used in this work are listed in Table 3. Yeast strains were propagated in 10 g/liter yeast extract, 20 g/liter peptone, and 20 g/liter d-glucose (YP and 2% glucose). For solid media, YP and 2% glucose was supplemented with 20 g/liter agar. Fermentation experiments were conducted in YP and 10% glucose. For selection of strains expressing the HPH or KanMX4 resistance marker, 300 mg/ml hygromycin B or 200 mg/ml Geneticin was added to the medium, respectively. For selection of haploid strains expressing the NatMX marker, 50 mg/ml was added; for diploid strains, 75 mg/ml was added. Strains were maintained at −80°C in stock medium composed of YP and 26% glycerol.

**TABLE 3:** Yeast strains used in this study

Yeast strain	Species	Main characteristics	Source or reference
ER18alpha	Saccharomyces cerevisiae	Haploid segregant of the first-generation bioethanol strain Ethanol Red (Fermentis/Lesaffre)	83
ER18A	Saccharomyces cerevisiae	Mating type switched ER18alpha	83
ER18A HPH	Saccharomyces cerevisiae	ER18A with an *HPH* resistance marker introduced at chrIV at position 867910 (genome version R64-2-1 S288C; SGD), *MAT***a**	This study
KEA1	Saccharomyces cerevisiae	Whole-genome transformant of ER18A HPH with DNA of K. marxianus CBS6014	This study
KEA2	Saccharomyces cerevisiae	Whole-genome transformant of ER18A HPH with DNA of K. marxianus CBS6014	This study
KEA9	Saccharomyces cerevisiae	Whole-genome transformant of ER18A HPH with DNA of K. marxianus CBS6014	This study
KEA17	Saccharomyces cerevisiae	Whole-genome transformant of ER18A HPH with DNA of K. marxianus CBS6014	This study
KEA21	Saccharomyces cerevisiae	Whole-genome transformant of ER18A HPH with DNA of K. marxianus CBS6014	This study
KEA24	Saccharomyces cerevisiae	Whole-genome transformant of ER18A HPH with DNA of K. marxianus CBS6014	This study
KEA26	Saccharomyces cerevisiae	Whole-genome transformant of ER18A HPH with DNA of K. marxianus CBS6014	This study
KEA28	Saccharomyces cerevisiae	Whole-genome transformant of ER18A HPH with DNA of K. marxianus CBS6014	This study
OEA26	Saccharomyces cerevisiae	Whole-genome transformant of ER18A HPH with DNA of *O. polymorpha* CBS5032	This study
OEA28	Saccharomyces cerevisiae	Whole-genome transformant of ER18A HPH with DNA of *O. polymorpha* CBS5032	This study
OEA34	Saccharomyces cerevisiae	Whole-genome transformant of ER18A HPH with DNA of *O. polymorpha* CBS5032	This study
OEA39	Saccharomyces cerevisiae	Whole-genome transformant of ER18A HPH with DNA of *O. polymorpha* CBS5032	This study
OEA40	Saccharomyces cerevisiae	Whole-genome transformant of ER18A HPH with DNA of *O. polymorpha* CBS5032	This study
OEA41	Saccharomyces cerevisiae	Whole-genome transformant of ER18A HPH with DNA of *O. polymorpha* CBS5032	This study
OEA46	Saccharomyces cerevisiae	Whole-genome transformant of ER18A HPH with DNA of *O. polymorpha* CBS5032	This study
OEA57	Saccharomyces cerevisiae	Whole-genome transformant of ER18A HPH with DNA of *O. polymorpha* CBS5032	This study
KEA17 *EAF1*^ER18A^	Saccharomyces cerevisiae	KEA17 *EAF1^KEA17^*::*EAF1*^ER18A^	This study
KEA17 *BSC5p*^ER18A^	Saccharomyces cerevisiae	KEA17 *BSC5p^KEA17^*::*BSC5p*^ER18A^	This study
KEA17 *tK(CUU)K*^ER18A^	Saccharomyces cerevisiae	KEA17 *tK(CUU)K^KEA17^*::*tK(CUU)K*^ER18A^	This study
KEA17 *NHA1p*^ER18A^	Saccharomyces cerevisiae	KEA17 *NHA1p^KEA17^*::*NHA1p*^ER18A^	This study
KEA24 *YHK8*^ER18A^	Saccharomyces cerevisiae	KEA24 *YHK8^KEA24^*::*YHK8*^ER18A^	This study
KEA24 *RTT10p*^ER18A^	Saccharomyces cerevisiae	KEA24 *RTT10p^KEA24^*::*RTT10p*^ER18A^	This study
KEA24 *HMX1p*^ER18A^	Saccharomyces cerevisiae	KEA24 *HMX1p^KEA24^*::*HMX1p*^ER18A^	This study
KEA24 *EMT2*^ER18A^	Saccharomyces cerevisiae	KEA24 *EMT2^KEA24^*::*EMT2*^ER18A^	This study
KEA24 *RPL36Bp*^ER18A^	Saccharomyces cerevisiae	KEA24 *RPL36Bp^KEA24^*::*RPL36Bp*^ER18A^	This study
OEA28 *PIS1*^ER18A^	Saccharomyces cerevisiae	OEA28 *PIS1^OEA28^*::*PIS1*^ER18A^	This study
OEA28 *CET1p*^ER18A^	Saccharomyces cerevisiae	OEA28 *CET1p^OEA28^*::*CET1p*^ER18A^	This study
OEA28 *ILV2*^ER18A^	Saccharomyces cerevisiae	OEA28 *ILV2^OEA28^*::*ILV2*^ER18A^	This study
OEA28 *MPH1*^ER18A^	Saccharomyces cerevisiae	OEA28 *MPH1^OEA28^*::*MPH1*^ER18A^	This study
OEA28 *trt2*^ER18A^	Saccharomyces cerevisiae	OEA28 *TRT2^OEA28^*::*trt2*^ER18A^	This study
ER18A HPH *tK(CUU)K*^KEA17^	Saccharomyces cerevisiae	ER18A HPH *tK(CUU)K^ER18A^*::*tK(CUU)K*^KEA17^	This study
ER18A HPH *EMT2*^KEA24^	Saccharomyces cerevisiae	ER18A HPH *EMT2^ER18A^*::*EMT2*^KEA24^	This study
ER18A HPH *TRT2*^OEA28^	Saccharomyces cerevisiae	ER18A HPH *trt2^ER18A^*::*TRT2*^OEA28^	This study
QD47	Saccharomyces cerevisiae	Diploid; cross of OEA28 and ER18alpha	This study
QD48	Saccharomyces cerevisiae	QD47 *TRT2/trt2*::*NatMX*	This study
BTC.1D	Saccharomyces cerevisiae	Haploid segregant of ale strain JT 22490; *MAT*α	(84)
K11	Saccharomyces cerevisiae	Acetic acid-tolerant sake brewing strain	Lab strain collection
GS1.11-26	Saccharomyces cerevisiae	Diploid, second-generation bioethanol production strain	(85)
CBS5032	Ogataea polymorpha	Haploid; high-temp growth on solid nutrient plates	CBS-KNAW
CBS6014	Kluyveromyces marxianus	Diploid; high-temp growth on solid nutrient plates	CBS-KNAW
CBS7555	Zygosaccharomyces bailii	Haploid; high acetic acid tolerance and moderate thermotolerance	CBS-KNAW
CBS732	Zygosaccharomyces rouxii	Haploid; highly osmotolerant and moderate thermotolerance	CBS-KNAW

### Molecular biology methods.

Yeast cells were transformed by electroporation (65) or with the LiAc/SS-DNA/PEG method (66). For whole-genome transformation, between 5 and 10 μg of gDNA was used as donor DNA. Yeast gDNA was extracted with the PCI method (67) and amplified with Q5 high-fidelity DNA polymerase (NEB) for sequencing purposes or with an in-house polymerase for diagnostic purposes. Correct integration of SNPs was verified via SNP PCR prior to Sanger sequencing. DNA was sent for Sanger sequencing to Eurofins (GATC, Germany).

### Spot test and small-scale fermentations.

For spot tests, strains were grown overnight and diluted to an optical density at 600 nm of 0.5. The cell suspensions were spotted onto YP and 2% glucose plates, together with a 10-fold dilution series, and incubated at the appropriate temperature and/or on YP and 2% glucose plates supplemented with 50 mg/ml nourseothricin, as appropriate. The images were converted to gray scale, and contrast and brightness were adjusted with Adobe Photoshop CS6 with the same settings for all panels.

For small-scale fermentations, strains were pregrown for 48h in 50 ml YP and 2% glucose. Semianaerobic fermentations were performed in cylindrical glass tubes (150 ml) sealed with a cotton-plugged rubber stopper. Yeast was pitched at 1 g (dry weight)/liter in a total volume of 60 ml YP and 10% glucose, followed by incubation at various temperatures ranging from 30 to 42°C. The medium was continuously stirred at 120 rpm using a magnetic stirrer. During fermentations, CO_2_ production correlates with the consumption of sugars and was measured to determine the progress of the fermentations.

### *E. coli* transformation and plasmid isolation.

Competent TOP10′ E. coli cells were prepared using the rubidium-chloride method (68). Plasmids were transformed in E. coli cells using heat shock (69). Plasmids were propagated overnight at 37°C in E. coli cells (Invitrogen) by incubation in liquid Luria-Bertani medium with 100 μg/ml ampicillin. Isolation of plasmids was performed using a Macherey-Nagel NucleoSpin plasmid kit according to the manufacturer’s instructions.

### CRISPR/Cas9 genome editing.

For each target gene to be modified, unique gRNAs were constructed and cloned into the gRNA plasmid’s backbone. The gRNA plasmid vector was cleaved with EcoRV and XhoI (NEB) according to the manufacturer’s instructions. A list of gRNAs can be found in Table S3. For intergenic regions, two gRNAs were cloned in the 2xgRNA plasmid to generate sites for double cutting. In this case, linear, PCR-amplified donor DNA containing two synonymous SNPs to avoid recutting of the DNA was used. To rule out side effects of these SNPs, the original SNP was reintroduced, and the strains were evaluated for thermotolerance. For SNPs in open reading frames (ORFs), duplexed DNA oligomers were used as donor. In a first transformation, the target strain was transformed with a plasmid containing *Sp*Cas9 and a KanMX4 marker. A second transformation introduced the donor DNA and the gRNA plasmid containing a NatMX marker. The transformants were plated on double selection medium and incubated at 30°C for 2 to 3 days. Integration of the correct SNP or gene was verified by Sanger sequencing. Plasmids were lost by sequential transfer of the strain in nonselective medium. Single cells were obtained using a micromanipulator (Singer MSM 300).

10.1128/mBio.03649-20.8 TABLE S3 List of gRNAs used in this study. The PAM site is indicated in boldface. NA, not applicable. Download Table S3, DOCX file, 0.03 MB. Copyright © 2021 Deparis et al. 2021 Deparis et al. https://creativecommons.org/licenses/by/4.0/ This content is distributed under the terms of the Creative Commons Attribution 4.0 International license.

### Tetrad analysis.

Diploid strains were suspended in water and plated on solid potassium acetate (1%) medium to allow sporulation. After 5 to 9 days, a small number of cells were suspended in 47 μl of sterile water to which 3 μl of lyticase (10,000 U/ml) was added, after which the mixture was incubated at room temperature for 3 min. Tetrads were picked and separated with the micromanipulator (Singer MSM 300). The images were converted to gray scale, and the contrast and brightness were adjusted with Adobe Photoshop CS6 with the same settings for all panels.

### Determination of ploidy by flow cytometry.

Briefly, exponentially growing cells (8 h) were washed with ice-cold sterile water and fixed with 70% ethanol at 4°C for at least 48 h. The DNA was stained with propidium iodide (0.046 M) in 50 mM Tris (pH 7.7) and 15 mM MgCl_2_ at 4°C for at least 72 h. The fluorescence intensity was measured using a FACScan instrument (Becton Dickinson) (70).

### Genomic DNA isolation and whole-genome sequencing.

Strains were grown in 50 ml of YP and 2% glucose for 2 days at 30°C. DNA was extracted using a MasterPure yeast DNA purification kit (Lucigen). About 30 μg of high-quality DNA was sent for sequencing to BGI (Hong Kong). Using the Illumina HiSeq2500 technology, 125-bp paired-end, 500-bp insert length libraries were generated. An average sequence coverage over 70× was generated for all strains.

### DNA mapping and identification of putative causal SNPs.

Paired-end reads were mapped using the NGSEP pipeline (version 3.3.1) (71). In brief, reads from S. cerevisiae, K. marxianus, and *O. polymorpha* were mapped using bowtie 2 (72, 73) against the reference genome of S288C version R64-2-1 (SGD), DMKU3-1042 (GenBank GCA_001417885.1) and NCYC495 leu1.1 (Joint Genome Institute), respectively. Variants were called with parameters [-runRP -runRep -runRD -maxBaseQS 30 -minQuality 40 -maxAlnsPerStartPos 2 -knownSTRs <STR_file>]. The STR file of each reference genome was generated with the Tandem Repeats Finder (74) using the recommended parameters. The combined .vcf file was filtered using parameter [-q 40] and annotated using NGSEP. In-house scripts were used to apply further filtering and identify variants between the genome of the whole-genome transformants and ER18A (synonymous SNPs were filtered out). To investigate non-S288C elements, nonmapped reads of ER18A were assembled *de novo* using CLC Genomics Workbench 10 (Qiagen) with default parameters (minimum contig length, 300 bp). This assembly was added to the S288C reference and subsequently used as a new reference, and the complete pipeline was repeated to identify possible variants and/or new genes. Alternatively, these contigs were blasted against a BLAST database of all K. marxianus CBS6014 or *O. polymorpha* CBS5032 reads. Regions surrounding the identified SNPs were blasted against the raw read data of both thermotolerant yeasts to exclude the presence of fragments of donor (DMKU3-1042 or NCYC495) DNA.

### tRNA stability calculations.

The sequence of the *TRT2*-encoded tRNA in strain S288C was downloaded from the gtRNA database (http://gtrnadb.ucsc.edu/), together with its structure annotation. For the ER18A and OEA28 allele, the required mutations were introduced and the resulting structures analyzed with RNAeval (75, 76) with parameters [-v –d2 –T <temperature>]. The temperature influence was assessed at 30, 35, and 42°C.

### Total Thr^ACG^ count and phosphorylated Thr^ACG^ count.

The complete proteome of S. cerevisiae was downloaded from SGD (www.yeastgenome.org). Codon usage was determined on https://www.bioinformatics.org/sms2/codon_usage.html, and the total number of Thr^ACG^ codons in the proteome was determined. We then cross-referenced the positions of these Thr^ACG^ codons to all known and putative phosphorylation sites in S. cerevisiae on the PhosphoGrid Database (now part of BioGrid [thebiogrid.org]).

### RNA isolation and RT-qPCR.

For RNA extraction, fermentations at 30 or 42°C were performed in cylindrical tubes with an extrusion, sealed with a membrane. First, 5 ml of the fermentation broth was taken after approximately 50% of all glucose was consumed (8 h at 30°C and 10 h at 42°C). The cell suspensions were immediately placed on ice and spun down for 10 min at 14,000 rpm. The supernatant was removed, and the cell pellet was stored at −80°C after freeze-drying in liquid nitrogen. RNA was extracted using the TRIzol method (Invitrogen), as previously described (77).

For conversion of RNA to cDNA, 2 μg of RNA was first treated with DNase at 37°C for 15 min. The enzyme was then deactivated for 10 min at 65°C. Half of this mixture was used to produce cDNA with the iScript cDNA kit from Bio-Rad (catalog no. 170-8891) according to the manufacturer’s instructions.

To compare RNA levels of the genes of interest during fermentation, a standard curve was first made to assess the efficiency of each primer pair using a SYBR Green assay (Thermo Fisher). A list of primers can be found in Table S4 in the supplemental material. For household genes and genes of interest, a GoTAq qPCR Master Mix kit from Promega (A6002) was used according to the manufacturer’s instructions. The PCRs were performed in a StepOnePlus real-time PCR apparatus (Bio-Rad) and analyzed with the 2^–ΔΔ^*^CT^* method. *TDH2* and *FBA1* were used as household genes for comparison. The fold change was calculated compared to ER18alpha as a reference sample.

10.1128/mBio.03649-20.9 TABLE S4 RT-qPCR primers. Download Table S4, DOCX file, 0.03 MB. Copyright © 2021 Deparis et al. 2021 Deparis et al. https://creativecommons.org/licenses/by/4.0/ This content is distributed under the terms of the Creative Commons Attribution 4.0 International license.

### Shotgun proteomics analysis.

For protein extraction, fermentations at 42°C were performed in cylindrical tubes with an extrusion, sealed with a membrane. Samples were taken in midexponential phase (∼10 h at 42°C). The amount of protein in a culture aliquot was determined by the Lowry method, and an equivalent of about 2 mg of protein in cell mass was taken for further analysis. The cell suspensions were immediately placed on ice and spun down for 10 min at 14,000 rpm. The supernatant was removed, and the cell pellet was washed twice with PBS and then stored at −80°C after freeze-drying in liquid nitrogen. Each strain was analyzed in triplicate by the VIB Proteomics Core (https://corefacilities.vib.be/pec).

The yeast cell pellet was dissolved in 8 M urea buffer (in 20 mM HEPES [pH 8.0]) and added to a 2-ml microcentrifuge tube containing Lysing Matrix Y (MP Biomedicals, CA). Cell disruption was carried out after transfer to cooled holders on a Tissue Lyser II instrument (Qiagen, Venlo, Netherlands) for 5 min at 30 Hz. Samples were then sonicated and spun down. Protein concentrations were measured on recovered supernatants, using the Bradford method, and from each sample 100 μg of protein was used to continue the protocol. Samples were diluted with 20 mM HEPES (pH 8.0) to a urea concentration of 4 M, and proteins were digested with 1 μg of LysC (Wako; 1/100, wt/wt) for 4 h at 37°C. Samples were further diluted to a urea concentration of 2 M and digested with 1 μg of trypsin (Promega; 1/100, wt/wt) overnight at 37°C. The resulting peptide mixture was acidified by addition of 1% trifluoroacetic acid (TFA) and, after a 15-min incubation on ice, samples were centrifuged for 15 min at 1,780 × *g* at room temperature to remove insoluble components. Next, the peptides were purified on OMIX C_18_ tips (Agilent). The tips were first washed three times with 200 μl prewash buffer (0.1% TFA in water/acetonitrile [ACN; 20:80, vol/vol]) and preequilibrated five times with 200 μl of solvent A (0.1% TFA in water/ACN [98:2, vol/vol]) before the samples were loaded on the tip. After peptide binding, the tip was washed three times with 200 μl of solvent A, and then the peptides were eluted twice with 150 μl of elution buffer (0.1% TFA in water/ACN [40:60, vol/vol]).

Purified peptides were redissolved in 50 μl of loading solvent A (0.1% TFA in water/ACN [98:2, vol/vol]), and the peptide concentration determined on a Lunatic spectrophotometer (Unchained Labs, Ghent, Belgium) (49). Next, 2 μg of peptide material of each sample was injected for LC-MS/MS analysis in an Ultimate 3000 RSLC nano LC (Thermo Fisher Scientific, Bremen, Germany) connected in-line to a Q Exactive HF mass spectrometer (Thermo Fisher Scientific) equipped with a nanospray flex ion source (Thermo Fisher Scientific).

Trapping was performed at 10 μl/min for 4 min in loading solvent A on a 20-mm trapping column (made in-house, 100-μm internal diameter, 5-μm beads, C_18_ Reprosil-HD; Dr. Maisch, Germany). The peptides were separated on a nanoEase MZ C18 HSS T3 column (100 Å, 1.8 μm, 75 μm × 250 mm; Waters). The column was kept at a constant temperature of 50°C. The peptides were eluted by a nonlinear gradient from 1 to 55% MS solvent B (0.1% TFA in water/ACN [20:80, vol/vol]) over 145 min, at a constant flow rate of 300 nl/min, followed by a 5-min washing phase plateauing at 99% MS solvent B. Reequilibration with 99% MS solvent A (0.1% TFA in water) was performed at 300 nl/min for 45 min The mass spectrometer was operated in data-dependent mode, automatically switching between MS and MS/MS acquisition for the 16 most abundant ion peaks per MS spectrum. Full-scan MS spectra (375 to 1,500 *m/z*) were acquired at a resolution of 60,000 (at 200 *m/z*) in an Orbitrap analyzer after accumulation to a target value of 3,000,000. The 16 most intense ions above a threshold value of 13,000 were isolated for fragmentation at a normalized collision energy of 28% after filling the trap at a target value of 100,000 for a maximum 80 ms. MS/MS spectra (200 to 2,000 *m/z*) were acquired at a resolution of 15,000 in the Orbitrap analyzer.

### Mass spectrometry data analysis.

Data analysis was performed with MaxQuant (version 1.6.11.0) using the Andromeda search engine with default search settings, including a false discovery rate (FDR) set at 1% on both the peptide and protein level (78, 79). Spectra were searched against the Saccharomyces cerevisiae (taxonomy ID 559292) UniProt reference proteome (www.uniprot.org; database version: January 2020 with 6,049 entries). The mass tolerance for precursor and fragment ions was set to 4.5 and 20 ppm, respectively, during the main search. Enzyme specificity was set to C-terminal arginine and lysine, also allowing cleavage next to prolines with a maximum of two missed cleavages. Variable modifications were set to oxidation of methionine residues, as well as acetylation of protein N termini. Matching between runs was enabled with a matching time window of 0.7 min and an alignment time window of 20 min. Only proteins with at least one unique or razor peptide were retained, leading to the identification of 2,604 proteins. Proteins and peptides were quantified by the MaxLFQ algorithm, integrated in the MaxQuant software. A minimum ratio count of two unique or razor peptides was required for quantification. Further data analysis was performed with the Perseus software (version 1.6.2.1) after uploading the protein groups and peptide files from MaxQuant (80). Reverse database hits were removed and replicate samples were grouped. LFQ intensities were log_2_ transformed for both proteins and peptides. Features with fewer than three valid values in at least one group were removed, and missing values were imputed from a normal distribution around the detection limit, resulting in 2,047 and 18.059 quantified proteins and peptides, respectively, which were subsequently used for further data analysis.

PCA was performed on the log_2_-transformed LFQ protein intensities using Perseus. To reveal proteins subjected to regulation in the host strain ER18A HPH and the three WG transformants OEA28, KEA17, and KEA24 relative to the control strain ER18alpha, quantified proteins were subjected to a two-sided, modified unpaired *t* test using as cutoff values a permutation-based FDR of 0.05 and an S_0_ background variance parameter of 1 (81). A heatmap of all regulated proteins across the four pairwise comparisons was prepared from a matrix of z-scored log_2_-transformed LFQ protein intensities. Proteins and samples were displayed according to the order of groups defined by hierarchical clustering using Euclidean distances as dissimilarity matrix and average linkage settings. GO terms of significantly up- or downregulated proteins were collected from SGD (www.yeastgenome.org) and grouped together according to their biological function identified in yeast.

Misincorporation of threonine in mutant strains was verified in two ways. On the one hand, we measured the fraction of threonine-to-lysine (T>K) and threonine-to-methionine (T>M) substitutions for each strain versus the control strain to check whether it was higher than 1. This analysis was performed on the peptide LFQ intensities from a second MaxQuant search in which T>K or T>M amino acid substitutions were set as variable modifications. On the other hand, a downward shift in the distribution of log_2_-fold change for threonine-containing peptides relative to the same distribution for all peptides was checked for the host strain and the three WG transformants versus the control strain.

### Relative RNA transcription rate and Thr^ACG^ abundance calculation after heat shock.

Genomic Run On data (46) were analyzed with GEO2R. Data sets from RNA-seq performed at 4, 11, 16, 26, and 40 min at 37°C were compared against 0 min at 37°C, which was used as the reference data set. The sequences of the top 25 most highly expressed genes were extracted from YeastMine (https://yeastmine.yeastgenome.org/yeastmine/begin.do), as well as the sequences of all genes tested. The codon usage was determined on the SMS codon usage webserver (https://www.bioinformatics.org/sms2/codon_usage.html), and the relative codon usage frequencies for each amino acid were calculated. We then took the ratio of the codon frequency of the average of the top 25 induced proteins versus the average of all proteins and plotted this for the various time points. In addition, we took the RNA polymerase II transcription rate data from Castells-Roca et al. (46). For the mRNA abundance calculations, we analyzed the difference in mRNA abundance of all proteins versus the same top 25 most transcribed genes for each time point. The normal distribution of the groups has been rejected by a D’Agostino and Pearson’s test (α = 0.05). The significance levels between all proteins and the top 25 most induced proteins were calculated for each time point with a Kruskal-Wallis test (α = 0.05).

### Identification of anticodon changes.

For identification of anticodon changes across 1,011 sequenced S. cerevisiae genomes, we analyzed the 1011Matrix.gvcf.gz (http://1002genomes.u-strasbg.fr/files/) file of all variants for base changes at the anticodon positions of all tRNAs based on the S. cerevisiae R64-1-1 genome assembly.

### Phylogenetic analysis of tRNA anticodon switches.

The genomic tRNA sequences of 70 archaeal, 576 bacterial, and 495 eukaryotic strains/species were downloaded from the gtRNA database. As an initial efficient method for identification of tRNA changes, the sequences of each strain were aligned to each other using bowtie2 (72, 73) with the parameters –no-unal –no-head and -a, the latter to retain all valid alignments. A custom script was written to select from the alignments obtained by bowtie2 and for each tRNA sequence the closest nonequal alignment and count the number of such alignments between tRNAs coding for different amino acids. For Xenopus tropicalis, Arabidopsis thaliana, Schizosaccharomyces japonicus, and Bacillus anthracis, the specific tRNA families were selected and aligned using CLC Genomics Workbench. A phylogenetic neighbor-joining tree was constructed with a bootstrap value of 100 in a Jukes-Cantor substitution model. The phylogenetic tree was visualized with SplitsTree4 (82).

### Data availability.

All sequence data have been submitted to the NCBI Sequence Read Archive (genome resequencing data, PRJNA575820). The mass spectrometry proteomics data have been deposited to the ProteomeXchange Consortium via the PRIDE (86) partner repository with the dataset identifier PXD024655.
