# Supplementary figures and images for: Whole-Genome Transformation Promotes tRNA Anticodon Suppressor Mutations under Stress
Source: mBio. 2021 Mar 23;12(2):e03649-20. doi: 10.1128/mBio.03649-20 (PMC8092322; doi:10.1128/mBio.03649-20)

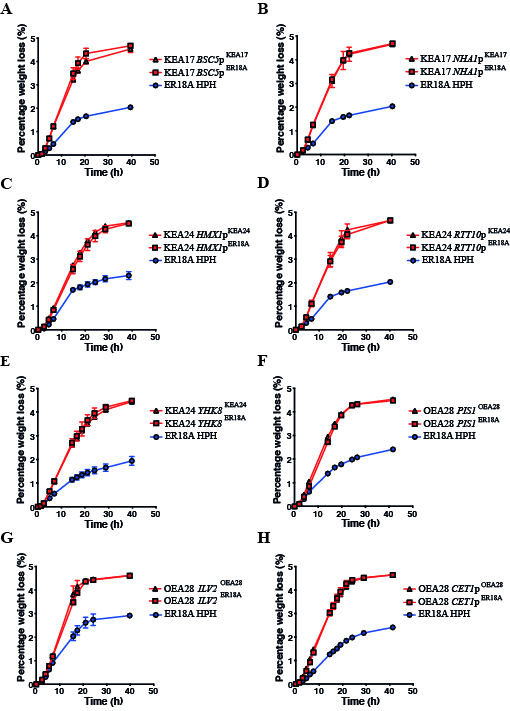

Supplement: FIG S1 [file mBio.03649-20-sf001.tif]

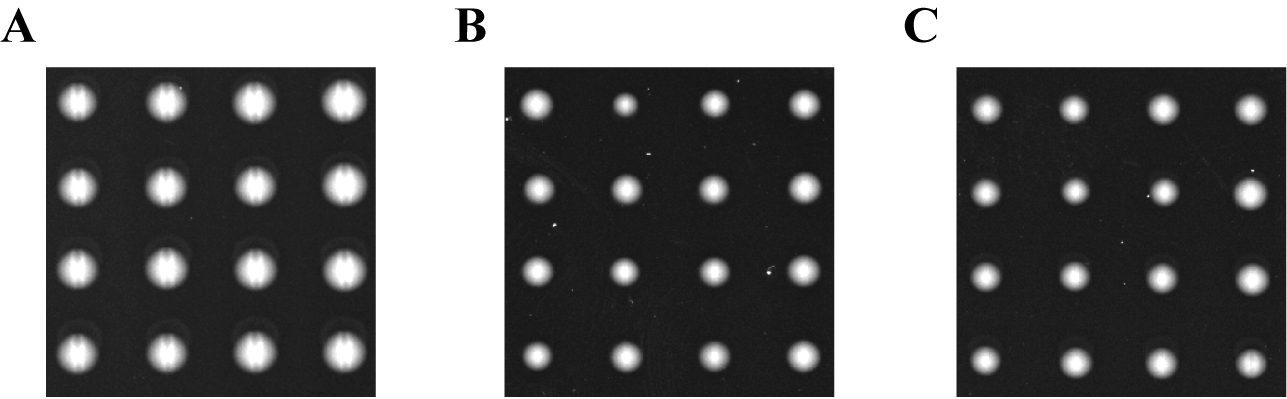

Supplement: FIG S2 [file mBio.03649-20-sf002.tif]

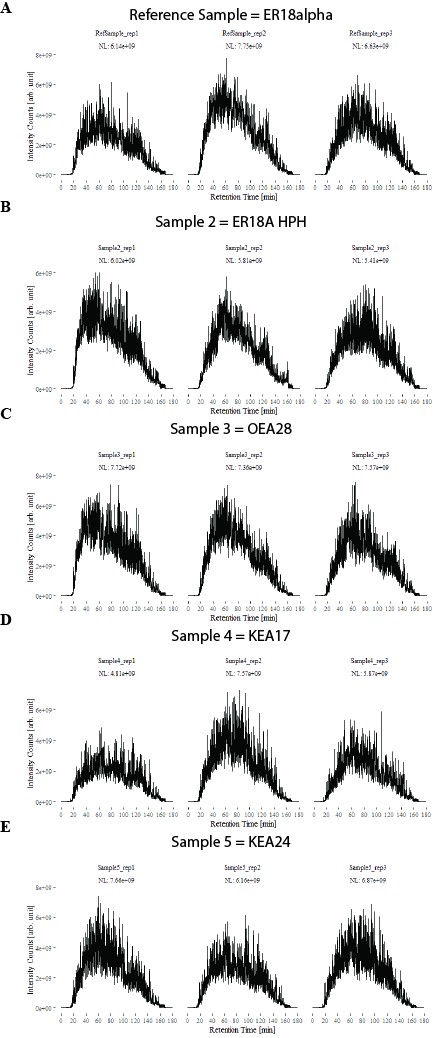

Supplement: FIG S3 [file mBio.03649-20-sf003.tif]

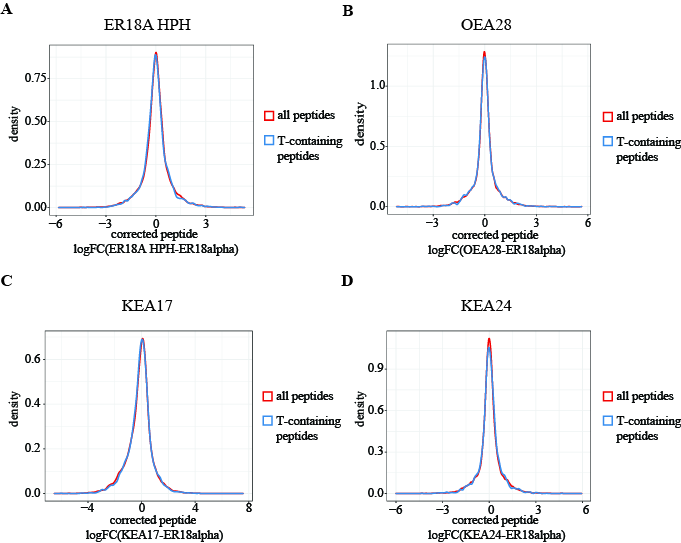

Supplement: FIG S4 [file mBio.03649-20-sf004.tif]

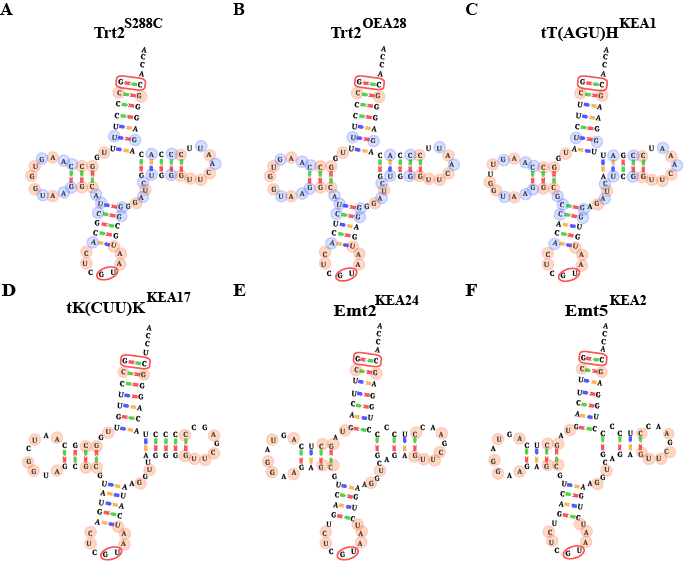

Supplement: FIG S5 [file mBio.03649-20-sf005.tif]
