# Supplementary material for: Whole-Genome Transformation Promotes tRNA Anticodon Suppressor Mutations under Stress
Source: mBio. 2021 Mar 23;12(2):e03649-20. doi: 10.1128/mBio.03649-20 (PMC8092322; doi:10.1128/mBio.03649-20)
Supplement: TABLE S1 [file mBio.03649-20-st001.docx]

**Table S1 GO terms and associated gene products.**

| **GO ID** | **Term** | **Genes** |
| --- | --- | --- |
| **Genes encoding more abundant proteins** | | |
| GO:0006520 | cellular amino acid metabolic process | *ACO2, ADI1, ARG4, ARG7, ARO1, ARO10, BNA1, HIS3, LYS1, MET13, SAM2, SRY1, STR3* |
| GO:0006364 | rRNA processing | *EFG1, ESF1, FAL1, KRR1, NOG1, NOP15, RPF1, RPF2, TSR2* |
| GO:0032787 | monocarboxylic acid metabolic process | *ALD5, BNA1, CRC1, ICL2, PDH1, SRY1, TMA17, YAT1, YAT2* |
| GO:0006629 | lipid metabolic process | *CRC1, ICL2, INO1, PDH1, TMA17, YAT1, YAT2* |
| GO:0007005 | mitochondrion organization | *ATP20, COA6, ERV1, MGM101, UTH1* |
| GO:0042273 | ribosomal large subunit biogenesis | *NOG1, NOP15, RLP24, RPF1, RPF2* |
| GO:0006811 | ion transport | *ATP20, CRC1, NCE102, PMP3* |
| GO:0042274 | ribosomal small subunit biogenesis | *EFG1, FAL1, KRR1, TSR2* |
| GO:0055085 | transmembrane transport | *ATP20, CRC1, ERV1, SIL1* |
| GO:0000054 | ribosomal subunit export from nucleus | *NOG1, NOG2, RPF1* |
| GO:0005975 | carbohydrate metabolic process | *INO1, UBC8, UTH1* |
| GO:0042221 | response to chemical | *EFG1, ERV1, PMP3* |
| GO:0051049 | regulation of transport | *NCE102, NOG2* |
| GO:0042255 | ribosome assembly | *RLP24, RPF2* |
| GO:0071554 | cell wall organization or biogenesis | *NCE102, UTH1* |
| GO:0006873 | cellular ion homeostasis | *COA6, ERV1* |
| GO:0006605 | protein targeting | *ERV1, SIL1* |
| GO:0055086 | nucleobase-containing small molecule metabolic process | *ATP20, BNA1* |
| GO:0006366 | transcription by RNA polymerase II | *SPT23* |
| GO:0006869 | lipid transport | *NCE102* |
| GO:0070647 | protein modification by small protein conjugation or removal | *UBC8* |
| GO:0006325 | chromatin organization | *SPT23* |
| GO:0006310 | DNA recombination | *MGM101* |
| GO:0000746 | conjugation | *EFG1* |
| GO:0006281 | DNA repair | *MGM101* |
| GO:0001403 | invasive growth in response to glucose limitation | *DIA1* |
| GO:0006457 | protein folding | *MPD2* |
| GO:0008033 | tRNA processing | *PUS1* |
| GO:0006468 | protein phosphorylation | *NCE102* |
| GO:0000910 | cytokinesis | *UTH1* |
| GO:0045333 | cellular respiration | *ACO2* |
| GO:0006974 | cellular response to DNA damage stimulus | *MGM101* |
| GO:0031399 | regulation of protein modification process | *NCE102* |
| GO:0006401 | RNA catabolic process | *EFG1* |
| GO:0051603 | proteolysis involved in cellular protein catabolic process | *UBC8* |
| GO:0006979 | response to oxidative stress | *ERV1* |
| GO:0051604 | protein maturation | *MAP1* |
| GO:0007124 | pseudohyphal growth | *DIA1* |
| GO:0009451 | RNA modification | *PUS1* |
| GO:0006091 | generation of precursor metabolites and energy | *RGI2* |
| **Genes encoding less abundant proteins** | | |
| GO:0042221 | response to chemical | *AHP1, GCY1, GRE3, HSP104, HSP12, HSP26, HSP30, HSP31, HSP42, IML2, MDG1, SGT2, SRX1, SSA3, SSA4* |
| GO:0005975 | carbohydrate metabolic process | *ENO1, EXG1, GCY1, GLK1, GPM2, GPP2, GRE3, HXK1, LEU2, OPI10, PGM2, TDH1, YPI1* |
| GO:0032787 | monocarboxylic acid metabolic process | *ACB1, ALD4, DLD3, ENO1, FDH1, GLK1, GPM2, HSP31, HXK1, LEU2, SPE2, TDH1* |
| GO:0006091 | generation of precursor metabolites and energy | *ADH4, ALD4, ENO1, GLK1, GPM2, HXK1, PGM2, RGI1, TDH1, YPI1* |
| GO:0006979 | response to oxidative stress | *AHP1, GCY1, GRE3, HSP104, HSP12, HSP30, HSP31, HSP42, SRX1* |
| GO:0055085 | transmembrane transport | *ADY2, GLK1, HSP30, HXK1, HXT3, SSA3, SSA4, VMA10, YRO2* |
| GO:0006457 | protein folding | *HSP104, HSP26, HSP31, HSP42, SGT2, SSA3, SSA4, STI1* |
| GO:0055086 | nucleobase-containing small molecule metabolic process | *ENO1, GLK1, GPM2, HXK1, PGM2, PHO8, SPE2, TDH1* |
| GO:0009408 | response to heat | *HSP104, HSP12, HSP26, HSP30, HSP42, SGT2, SSA4* |
| GO:0006629 | lipid metabolic process | *ACB1, AYR1, ERG3, GPI8, NTE1, OPI10, TGL1* |
| GO:0006970 | response to osmotic stress | *GPP2, GRE3, HSP104, HSP12, HSP26, HSP30, HSP42* |
| GO:0006811 | ion transport | *ADY2, HSP30, HXT3, VMA10, YRO2* |
| GO:0071554 | cell wall organization or biogenesis | *EXG1, HSP150, PST1, SMI1* |
| GO:0006605 | protein targeting | *SGT2, SSA3, SSA4, STI1* |
| GO:0006281 | DNA repair | *DDR48, DEF1, RFA1, RFA2* |
| GO:0051321 | meiotic cell cycle | *ADY2, RFA1, RFA2, WTM1* |
| GO:0032200 | telomere organization | *DEF1, RFA1, RFA2* |
| GO:0006470 | protein dephosphorylation | *IGO1, PHO8, YPI1* |
| GO:0051603 | proteolysis involved in cellular protein catabolic process | *DEF1, SGT2, SSA3* |
| GO:0051726 | regulation of cell cycle | *IGO1, SMI1, YPI1* |
| GO:0048285 | organelle fission | *RFA1, RFA2, YPI1* |
| GO:0070647 | protein modification by small protein conjugation or removal | *DEF1, RFA1, RFA2* |
| GO:0006520 | cellular amino acid metabolic process | *ADH4, ARG3, LEU2* |
| GO:0008643 | carbohydrate transport | *GLK1, HXK1, HXT3* |
| GO:0006366 | transcription by RNA polymerase II | *HMLALPHA2, MATALPHA2, WTM1* |
| GO:0006873 | cellular ion homeostasis | *PGM2, VMA10, YPI1* |
| GO:0033043 | regulation of organelle organization | *ENO1, WTM1, YPI1* |
| GO:0000278 | mitotic cell cycle | *IGO1, SMI1, YPI1* |
| GO:0006974 | cellular response to DNA damage stimulus | *DEF1, HSP30* |
| GO:0009311 | oligosaccharide metabolic process | *HSP104, PGM2* |
| GO:0006401 | RNA catabolic process | *IGO1, JSN1* |
| GO:0048284 | organelle fusion | *ENO1, YKT6* |
| GO:0006766 | vitamin metabolic process | *SPE2, THI20* |
| GO:0007005 | mitochondrion organization | *STI1, YME2* |
| GO:0031399 | regulation of protein modification process | *IGO1, YPI1* |
| GO:0006310 | DNA recombination | *RFA1, RFA2* |
| GO:0007033 | vacuole organization | *ENO1, YKT6* |
| GO:0006260 | DNA replication | *RFA1, RFA2* |
| GO:0032543 | mitochondrial translation | *RML2* |
| GO:0007059 | chromosome segregation | *YPI1* |
| GO:0006325 | chromatin organization | *WTM1* |
| GO:0002181 | cytoplasmic translation | *RPL33A* |
| GO:0051604 | protein maturation | *MAK10* |
| GO:0006397 | mRNA processing | *YME2* |
| GO:0006417 | regulation of translation | *ANB1* |
| GO:0042274 | ribosomal small subunit biogenesis | *DBP8* |
| GO:0007010 | cytoskeleton organization | *HSP42* |
| GO:0048193 | Golgi vesicle transport | *YKT6* |
| GO:0000746 | conjugation | *MDG1* |
| GO:0043543 | protein acylation | *MAK10* |
| GO:0042273 | ribosomal large subunit biogenesis | *RPL33A* |
| GO:0006364 | rRNA processing | *DBP8* |
| GO:0018193 | peptidyl-amino acid modification | *MAK10* |
| GO:0006414 | translational elongation | *ANB1* |
| GO:0061025 | membrane fusion | *YKT6* |
| GO:0006497 | protein lipidation | *GPI8* |
| GO:0016050 | vesicle organization | *YKT6* |
