# Supplementary material for: Whole-Genome Transformation Promotes tRNA Anticodon Suppressor Mutations under Stress
Source: mBio. 2021 Mar 23;12(2):e03649-20. doi: 10.1128/mBio.03649-20 (PMC8092322; doi:10.1128/mBio.03649-20)
Supplement: TABLE S2 [file mBio.03649-20-st002.docx]

**Table S2 Anticodon mutations and number of other SNPs in the genome of additional heat-tolerant WG transformants**

| **Strain** | **Anticodon-mutated tRNA** | **Anticodon mutation** | **Number of other SNPs** |
| --- | --- | --- | --- |
| KEA1 | *tT(AGU)H* | AGU > CGU | 3 |
| KEA2 | *EMT5* | CAU > CGU | 6 |
| KEA28 | *EMT5* | CAU > CGU | 13 |
| OEA26 | *EMT5* | CAU > CGU | 3 |
| OEA34 | *EMT5* | CAU > CGU | 1 |
| OEA39 | *EMT5* | CAU > CGU | 5 |
| OEA57 | - | - | 3 |
