# Supplementary material for: Whole-Genome Transformation Promotes tRNA Anticodon Suppressor Mutations under Stress
Source: mBio. 2021 Mar 23;12(2):e03649-20. doi: 10.1128/mBio.03649-20 (PMC8092322; doi:10.1128/mBio.03649-20)
Supplement: TABLE S3 [file mBio.03649-20-st003.docx]

**Table S3 List of gRNAs used in this work.** The PAM site is indicated in bold. N/A: not applicable

| ***GENE*** | **gRNA 1** | **gRNA 2** |
| --- | --- | --- |
| ***EAF1*** | GTCAAAGCATTGTGAGCTCT**CGG** | N/A |
| ***NHA1*** | GGTTTATGATATCGTTGACC**AGG** | GGAGCAACTAGAAGTCTCAA**AGG** |
| ***BSC5*** | GGAGGTTCCTCCAAAGAGTG**TGG** | ACAAAATATCGCCAAAGGTT**TGG** |
| ***tK(CUU)K*** | TTCTAAGTGATCATATTTCG**TGG** | TGTACCAACAGTTGCTTATA**AGG** |
| ***YHK8*** | GCCGTCATTTTTTCATTCAC**TGG** | N/A |
| ***HMX1*** | TAAAATAACCGCAAAAATGG**AGG** | TCATCATGTTCTTTGCAGAA**AGG** |
| ***RTT10*** | AGTGCCTAAAGGCCCTGTTG**AGG** | ATTATGTTCTAGCTGGTTAT**GGG** |
| ***RPL36B*** | CTTTGGGGCTGGAGTCATTT**GGG** | GAGATTCTAAGGCTAAAGGG**AGG** |
| ***EMT2*** | GCTGCATCTCGACCAAGATT**GGG** | TAGCTTCGTGCTTGTAGCAG**CGG** |
| ***PIS1*** | ATTGGGGATACTGCATACAA**AGG** | N/A |
| ***ILV2*** | ATACCCATACGTTTATATGG**AGG** | N/A |
| ***MPH1*** | AATAACATGGTATTGAGTTT**GGG** | N/A |
| ***CET1*** | ATCTCAAAGTTTTAATCCTT**TGG** | AGCTTTATCGTTAGACGATC**TGG** |
| ***TRT2*** | CAAAACACTATCACATTTTC**AGG** | CTATGGAAGTCATAGTAGTG**AGG** |
