# Supplementary material for: Whole-Genome Transformation Promotes tRNA Anticodon Suppressor Mutations under Stress
Source: mBio. 2021 Mar 23;12(2):e03649-20. doi: 10.1128/mBio.03649-20 (PMC8092322; doi:10.1128/mBio.03649-20)
Supplement: TABLE S4 [file mBio.03649-20-st004.docx]

**Table S4 RT-qPCR primers**

| **Primer** | **Sequence 5’-3’** |
| --- | --- |
| FBA1-fw | GACTTGTACACCAAGCCAGA |
| FBA1-rv | GATGTCACCAGCGTACAAAC |
| TDH2-fw | AGACTGTTGACGGTCCATCC |
| TDH2-rv | CCTTAGCAGCACCGGTAGAG |
| TRT2-fw | TTTGGCCAAGTGGTAAGGCA |
| TRT2-rv1 | AATTGAACCCACGATCCCCA |
| TRT2-rv2 | AATTGAACCCACGATCCCCG |
| tRNA^Lys^_CUU_-fw | CTTGTTGGCGCAATCGGTAG |
| tRNA^Lys^_CUU_-rv | GGGCTCGAACCCCTAACCTT |
| tRNA^eMet^_CAU_-fw | GCTCAGTAGGAAGAGCGTCA |
| tRNA^eMet^_CAU_-rv | GGTTCGAACTCTCGACCTTCA |
| MET22-fw | AGGCCAGACATACTGCAAAC |
| MET22-rv | GGCAATTACGAAGGTGGTAGAA |
| RAT1-fw | GCATCCCTAGCACTCCTAAATC |
| RAT1-rv | GACCACGCAAGGTACTTGTTA |
| XRN1-fw | CTTCCCAGTAGCGTCAGTAATC |
| XRN1-rv | GTGTCCAAGAACATGCACTTAC |
| HAC1_u_-fw | CTTGGCTATCCCTACCAACTTC |
| HAC1_u_-rv | TGAGCAGCTCTTCTGTTTCTC |
| HAC1_i_-fw | GCGACGATATAGCGGGAAA |
| HAC1_i_-rv | AAACCTGACTGCGCTTCT |
| HSP104-fw | GATGATCAGGGTCGATTGTTCT |
| HSP104-rv | CCTTCATCGTACCCGACATAAC |
| SSA3-fw | CGGAAACCTTCTCTACCTATGC |
| SSA3-rv | AGGCGGAATGCCACTTAAT |
